# The *Cryptococcus neoformans* Alkaline Response Pathway: Identification of a Novel Rim Pathway Activator

**DOI:** 10.1371/journal.pgen.1005159

**Published:** 2015-04-10

**Authors:** Kyla S. Ost, Teresa R. O’Meara, Naureen Huda, Shannon K. Esher, J. Andrew Alspaugh

**Affiliations:** Departments of Medicine/ Molecular Genetics & Microbiology, Duke University School of Medicine, Durham, North Carolina, United States of America; University of Minnesota, United States of America

## Abstract

The Rim101/PacC transcription factor acts in a fungal-specific signaling pathway responsible for sensing extracellular pH signals. First characterized in ascomycete fungi such as *Aspergillus nidulans* and *Saccharomyces cerevisiae*, the Rim/Pal pathway maintains conserved features among very distantly related fungi, where it coordinates cellular adaptation to alkaline pH signals and micronutrient deprivation. However, it also directs species-specific functions in fungal pathogens such as *Cryptococcus neoformans*, where it controls surface capsule expression. Moreover, disruption of the Rim pathway central transcription factor, Rim101, results in a strain that causes a hyper-inflammatory response in animal infection models. Using targeted gene deletions, we demonstrate that several genes encoding components of the classical Rim/Pal pathway are present in the *C*. *neoformans* genome. Many of these genes are in fact required for Rim101 activation, including members of the ESCRT complex (Vps23 and Snf7), ESCRT-interacting proteins (Rim20 and Rim23), and the predicted Rim13 protease. We demonstrate that in neutral/alkaline pH, Rim23 is recruited to punctate regions on the plasma membrane. This change in Rim23 localization requires upstream ESCRT complex components but does not require other Rim101 proteolysis components, such as Rim20 or Rim13. Using a forward genetics screen, we identified the *RRA1* gene encoding a novel membrane protein that is also required for Rim101 protein activation and, like the ESCRT complex, is functionally upstream of Rim23-membrane localization. Homologs of *RRA1* are present in other *Cryptococcus* species as well as other basidiomycetes, but closely related genes are not present in ascomycetes. These findings suggest that major branches of the fungal Kingdom developed different mechanisms to sense and respond to very elemental extracellular signals such as changing pH levels.

## Author Summary

Microorganisms that cause human disease use various cues to determine when they encounter a host. One of these signals is the slightly alkaline pH of human tissues. We have defined components of a pH-responsive signaling pathway in the human fungal pathogen *Cryptococcus neoformans*. This particular signaling pathway is specific for fungi and important for the virulence of several plant and animal pathogens. We have identified a previously unknown protein that activates this pH-sensing pathway. This protein and its downstream signaling pathway play a major role in the interaction of this fungal pathogen and the host immune system.

## Introduction

To survive the harsh environment of the infected host, microorganisms must be able to sense their external environment and adaptively alter their cellular processes. For example, microorganisms often encounter changes in extracellular pH when moving between different microenvironments. For fungal pathogens, the alkaline-response transcription factor Rim101 plays a central role in adapting to changing environmental and host conditions.

Rim101 was first implicated in fungal pathogenesis in the human fungal pathogen *Candida albicans* in which the *rim101Δ* mutant was demonstrated to be defective in the yeast to hyphal transition, and therefore unable to cause disseminated infection [1]. More recently, Bertuzzi and Schrettl et al. demonstrated that the opportunistic pathogen *Aspergillus fumigatus* requires the Rim101 ortholog PacC for virulence in a murine model of infection [2]. Since then, several investigators have demonstrated that other human, insect, and plant fungal pathogens require this conserved alkaline response pathway to cause disease [3–7].

More recently, the physiological role of Rim101 has been defined in the opportunistic fungal pathogen, *Cryptococcus neoformans*. *C*. *neoformans* is a basidiomycete yeast that causes life-threatening meningitis in immunocompromised individuals. Due to the emergence of HIV and the increased use of immunosuppressive therapies, this once-rare pathogen has become a significant global health problem, with over 1 million new infections estimated each year [8]. In *C*. *neoformans*, Rim101 is required for the proper formation of the protective polysaccharide capsule, as well as growth under several stress conditions such as low iron, elevated salt concentrations, and alkaline pH [9,10]. In addition, Rim101 mediates cell wall modifications that allow for capsule attachment and the masking/repression of cell surface pathogen associated molecular patterns (PAMPs). In the *rim101Δ* mutant strain, failed cell wall masking of immunogenic PAMPs results in a dramatic hyper-inflammatory response and immune-mediated host damage in a mouse inhalation model of cryptococcal infection [11].

**Fig 1:**
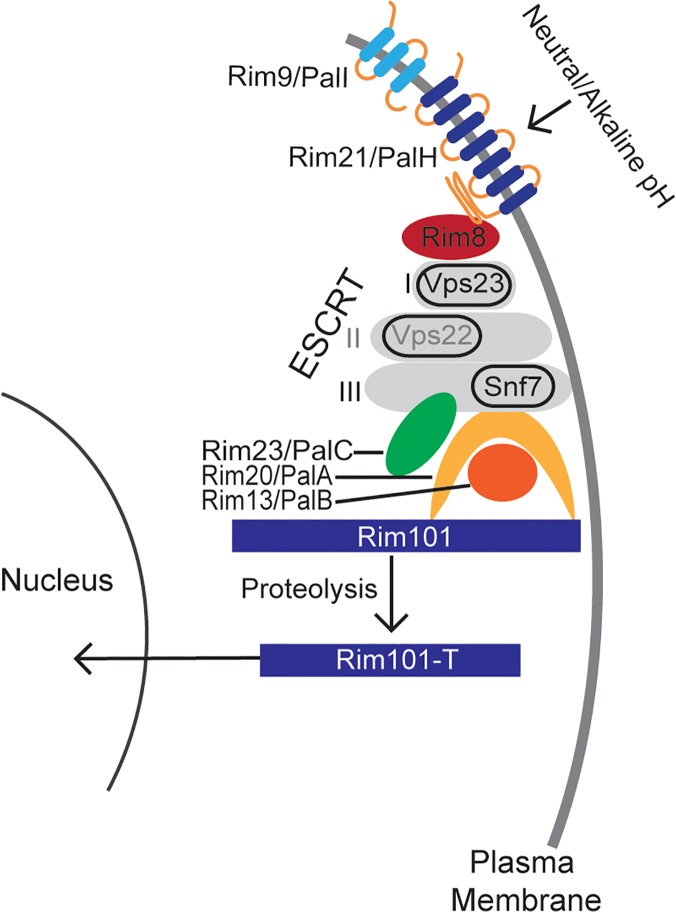
A model of the canonical Rim pathway elucidated in ascomycete fungi [21,24].

The signaling pathway responsible for sensing and activating Rim101 has been defined in model ascomycetes, such as *Aspergillus nidulans* and *Saccharomyces cerevisiae*, and it is highly conserved throughout other members of this phylum (Fig 1). In these species, initial activation of the Rim/Pal pathway involves extracellular sensing of pH by the 7-transmembrane domain receptor, Rim21/PalH [12–14]. This protein is a component of the membrane sensing complex, also comprised of the arrestin-like proteins Rim8/PalF, as well as the chaperone Rim9/PalI proteins [12,13,15]. Several ascomycete fungi require an additional component of the membrane signaling complex, Dfg16, which may serve as a Rim21/PalH chaperone [16]. Rim8/PalF recruitment to the sensing complex results in activation of part of the ESCRT complex, proteins also involved in endosome function [17–19]. Rim8/PalF binding to the ESCRT-I protein Vps23 induces the assembly of the ESCRT-II and—III complexes as a scaffolding platform for Rim pathway activation [19,20]. On this platform, the Rim proteolysis complex forms, composed of Rim23/PalC, Rim20/PalA, and the Rim13/PalB protease [21]. The intact Rim proteolysis complex catalyzes cleavage and activation of its target, the Rim101/PalC transcription factor [22,23]

This Rim/Pal signaling pathway has only recently been analyzed in basidiomycetes, a diverse group of fungi including some human and animal pathogens [9,25]. In the maize pathogen *Ustilago maydis*, the ESCRT-interacting complex (Rim23/PalC Rim20/PalA, and Rim13/PalB) was conserved and required for Rim101/PacC activation. However, elements of the membrane receptor complex (Rim9/PalI, Rim21/PalH, and Rim8/PalF) were either missing from the genome or not required for Rim101 processing [26]. In *C*. *neoformans*, there are several differences in the mechanism of Rim101 activation from established signaling paradigms in other fungal species. First, similar to *U*. *maydis*, the Rim/Pal membrane sensing complex proteins are not evident by homology searches. Also, we previously observed a novel connection between Rim101 and the cAMP/Protein Kinase A pathway [3,9]. The connection between these two conserved pathways reveals that the processes leading to Rim101 activation in this fungal species may occur in a somewhat distinct manner from that in distantly related ascomycetes. However, *C*. *neoformans* Rim101 activation also appears to require members of the canonical Rim activation pathway because deletion of the *RIM20* ortholog resulted in phenotypes identical to a *rim101Δ* mutation [9]. This observation implicated the involvement of both the cAMP and the classical pH response pathway in the activation of *C*. *neoformans* Rim101 [3,9]. While the *C*. *neoformans* cAMP/PKA signaling pathway has been extensively characterized, comparatively little is known about the *C*. *neoformans* Rim pathway

In this study, we determined that *C*. *neoformans* encodes functional orthologs of the major components of the Rim101 proteolysis complex, Rim13/PalB, Rim23/PalC, and Rim20/PalA. In contrast to these highly conserved distal components of Rim101 activation, the surface pH-responsive proteins, Rim8/PalF and Rim21/PalH, could not be identified using sequence or domain-based searches. Utilizing a random mutagenesis screen specifically designed to find *C*. *neoformans* Rim101 activators, we identified a previously unrecognized membrane protein required for *C*. *neoformans* Rim101 activation, which we named *RRA1* (Required for Rim101 Activation 1). While the Rra1 protein lacks significant sequence similarity to any component of the ascomycete Rim/Pal pathway, its predictive protein structure, containing 7-transmembrane domains, is similar to the Rim21/PalH sensors in ascomycetes. Moreover, predicted orthologs for Rra1 were found only in basidiomycetes. We also observe that Rim pathway signaling likely occurs in punctate regions on the plasma membrane. The recruitment of Rim23 to these puncta requires components of the ESCRT complex in addition to Rra1, suggesting that Rra1 functions upstream of other *C*. *neoformans* Rim pathway components. These findings suggest fundamental biological differences between the ways in which ascomycetes and basidiomycetes have developed cell surface signaling units to respond to common environmental signals.

## Results

### Neutral/alkaline pH induces Rim101 proteolytic cleavage and nuclear localization

**Fig 2:**
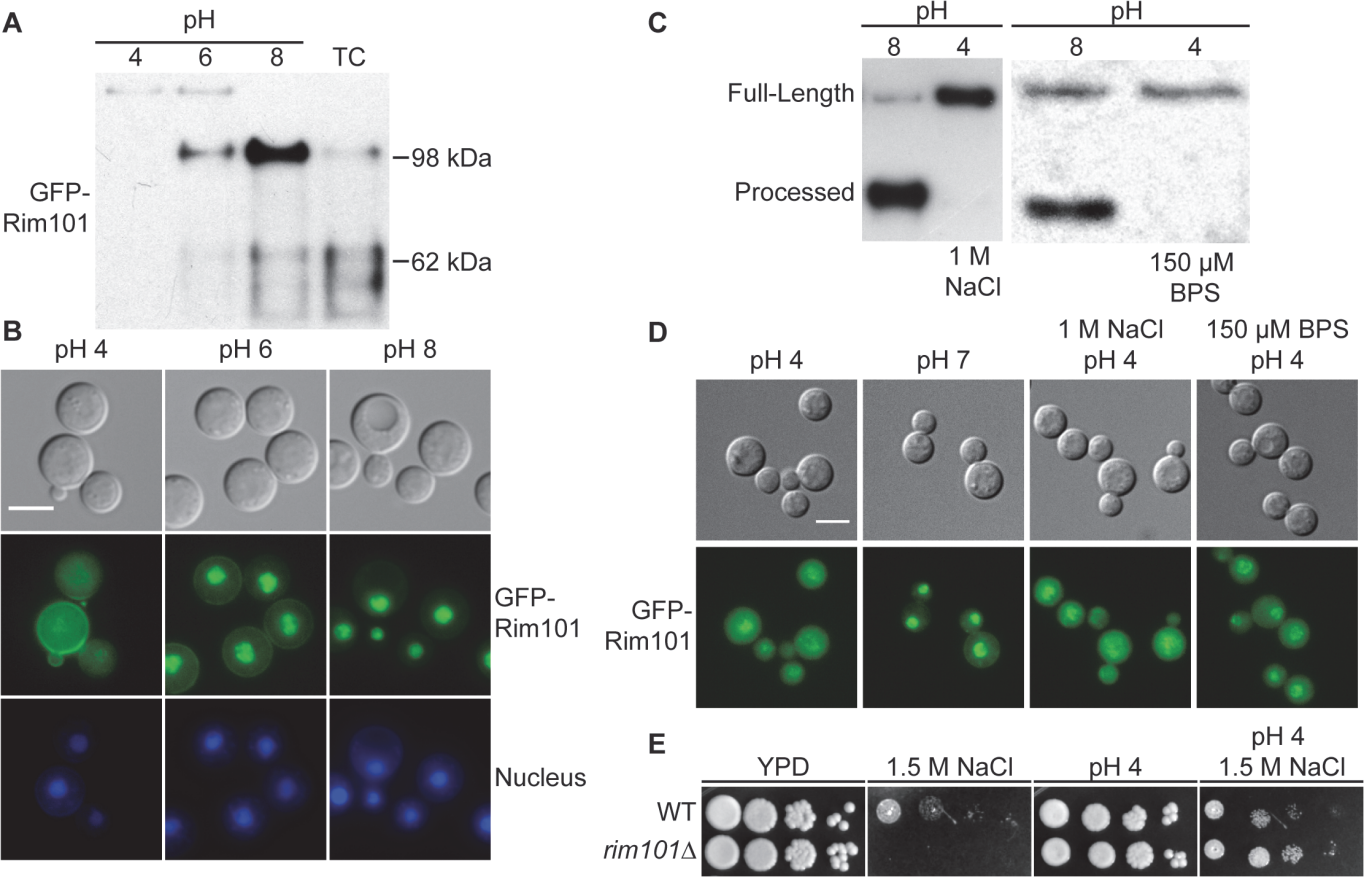
Rim101 proteolysis and nuclear localization are dependent on pH. (A) GFP-Rim101 is proteolytically processed from 140 kDa to ~100 kDa in response to increasing pH. GFP-Rim101 was immunoprecipitated from wild-type cells after incubating for 5 hr at the indicated pH 8 (SC medium buffered with McIlvaine’s buffer). Protein processing was determined by western blotting using an α-GFP antibody. (B) GFP-Rim101 nuclear localization increases in response to increasing pH. Cells were cultured in the same way as in (A). GFP signal was assessed by epifluorescence microscopy. Nuclei were stained using Hoechst 33342 live nuclei stain. Scale bar = 5 μm. (C) GFP-Rim101 proteolysis is not induced by 1 M NaCl or 150 μM BPS. Cells were cultured in each indicated condition for 3 hr. GFP-Rim101 was analyzed by western blot. (D) GFP-Rim101 localization in response to pH 7 SC (McIlvaine’s) or pH 4 SC (McIlvaine’s) with 1 M NaCl or 150 μM BPS. Cell assessed epifluorescence microscopy after 30 min incubation in each condition. Scale bar = 5 μm (E) *rim101Δ* is not NaCl sensitive at pH 4. Strains spotted onto YPD, YPD 150mM HEPES pH 4 1.5 M NaCl, and YPD 1.5 M NaCl.

As modeled in Fig 1, the Ascomycete Rim101/PacC transcription factor is activated by Rim13/PalB-mediated proteolytic cleavage in response to elevated pH signals. Previously, we demonstrated that the GFP-Rim101 fusion protein, when overexpressed by the histone *HIS3* promoter, was proteolytically cleaved from a 120 kDa form to a 70 kDa form in response to incubation in tissue culture medium [9]. To dissect the role of pH in Rim101 activation, we created a strain expressing GFP-Rim101 from its endogenous locus instead of the highly active histone3 promoter. The endogenously expressed *GFP-RIM101* allele fully rescued all *rim101Δ* mutant phenotypes. Using this strain, we assessed GFP-Rim101 proteolysis in defined media at pH 4, 6, and 8. At pH 4 we exclusively observed the intact 140 kDa form of the GFP-Rim101 protein, suggesting that this is a non-inducing condition for Rim101 proteolysis and activation. In a dose-response relationship with increasing pH, we observed increased proteolytic processing of GFP-Rim101 resulting in a predominant 100 kDa processed form as well as lower molecular weight forms that either represent further processing or proteolytic degradation (Fig 2A). In addition, GFP-Rim101 proteolytic processing correlated with increased nuclear localization (Fig 2B). Together, this demonstrates that the *C*. *neoformans* Rim101 protein is activated by increasing pH.

### Rim101 is not activated by high NaCl or iron limitation

In addition to responding to alkaline pH, *C*. *neoformans* Rim101 is required for growth in the virulence-relevant conditions of iron limitation and high salt concentrations [9]. These stress responses are linked with increasing pH because alkaline pH both reduces the bioavailability of iron and disrupts the membrane charge gradient necessary for ion channels to properly function [27].To determine whether Rim101 could also be activated by high salt concentrations and/or iron limitation independent of pH, we analyzed Rim101 activation in response to high salt and low iron conditions at pH 4. Neither GFP-Rim101 proteolytic processing nor nuclear localization was induced by the addition of 1 M NaCl (Fig 2C and 2D). Similarly, iron deprivation due to the addition of the iron chelator BPS only slightly increased GFP-Rim101 nuclear localization, but it did not induce Rim101 proteolytic processing (Fig 2C and 2D). In addition, the *rim101Δ* mutant did not have a growth defect on 1.5 M NaCl at pH 4 (Fig 2E), whereas it displays a salt sensitive phenotype at more alkaline pH. Therefore, *C*. *neoformans* Rim101 appears to be activated specifically by neutral/alkaline pH, rather than by other cell stress conditions such as salt stress or limiting iron availability.

### Identification and characterization of *C. neoformans* Rim13 and Rim23 orthologs

The signaling pathway responsible for Rim101 activation is highly conserved throughout fungi in the Ascomycota phylum (Fig 1). These proteins are typically recognizable by sequence conservation, even among fungi as divergent as the budding yeast *S*. *cerevisiae* and the hyphal fungus *A*. *nidulans*. In a previous study, we identified the gene encoding a functional ortholog of the Rim101 scaffolding protein Rim20 in the basidiomycete fungus *C*. *neoformans* and confirmed its role in capsule regulation, alkaline pH adaptation, and response to low iron [9]. Using *S*. *cerevisiae* and *A*. *nidulans* amino acid sequences for comparisons, we identified single genes encoding other components of the *C*. *neoformans* Rim101 proteolysis complex, including the Rim13 protease (CNAG_05601) and the Rim23 ESCRT-interacting protein (CNAG_02205) (S1 Fig).

**Fig 3:**
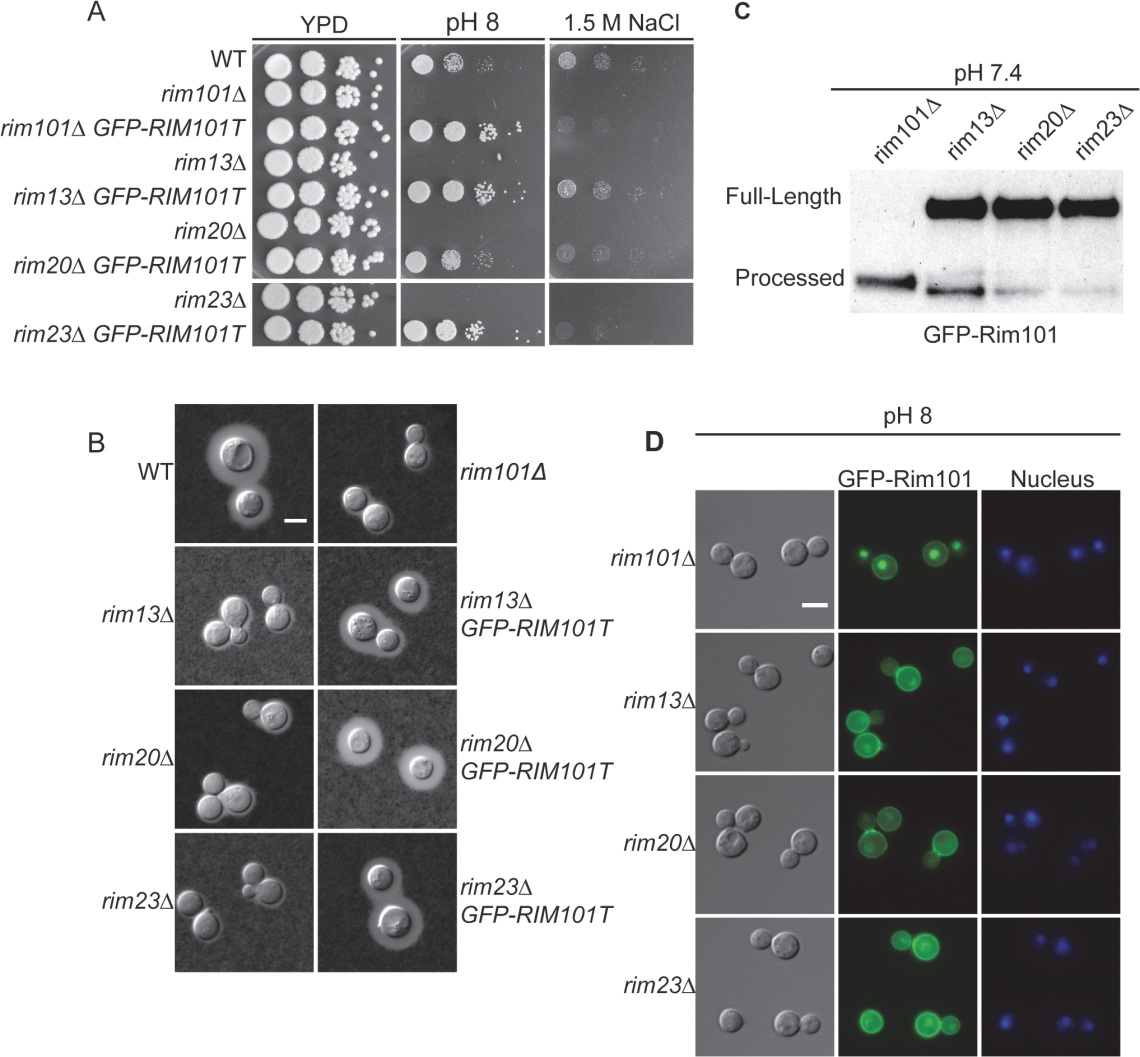
Role of Rim13 and Rim23 orthologs in Rim101-regulated phenotypes. (A) The *C*. *neoformans RIM13* and *RIM23* orthologs are required for pH 8 and NaCl tolerance. 10-fold serial dilutions of the indicated strains were spotted onto YPD, YPD 150mM HEPES pH 8, YPD 1.5M NaCl and incubated at 30^°^C for 48 hr -72 hr (B) The *rim13Δ* and *rim23Δ* mutants have a *rim101Δ-*like capsule defect. Cells were incubated in CO_2_-independent media for 48hr at 37^°^C. Capsule was visualized by counterstaining with India ink. (C) Rim101 proteolysis and localization are disrupted in *rim13Δ*, *rim20Δ*, and *rim23Δ* mutant strains. GFP-Rim101 was immunoprecipitated from each strain after 5 hr incubation in pH 7.4 YPD buffered with 150 mM HEPES. (D) GFP-Rim101 localization was assessed in the indicated strains after culturing for 5 hr in SC medium buffered with McIlvaine’s buffered to pH 8. Nuclei were stained with Hoechst 33342 live nuclei stain. Scale bar = 5 μm.

As expected, none of these genes were essential. Disruption of the *RIM13* and *RIM23* genes resulted in decreased growth on pH 8 and 1.5 M NaCl, similar to the *rim101Δ* mutant (Fig 3A). Notably, the *rim13Δ* and *rim23Δ* mutants also had a *rim101Δ-*like capsule defect, confirming a role for the pH-responsive signaling pathway in capsule regulation (Fig 3B). These *rim13Δ* and *rim23Δ* mutant phenotypes were rescued by reintroduction of the respective wild types alleles (S1 Fig).

To directly determine whether Rim13, Rim23, and Rim20 are upstream activators of Rim101, we assessed Rim101 proteolytic activation and nuclear localization in each mutant. All three genes were required for both GFP-Rim101 proteolysis and nuclear localization, demonstrating that Rim101 activation requires each predicted Rim pathway component (Fig 3C and 3D). In *A*. *nidulans* and *S*. *cerevisiae*, expression of an active Rim101/PacC protein, lacking its inhibitory C-terminal domain rescues mutations in upstream Rim pathway components [28,29]. In a similar manner, we expressed of a truncated form of *C*. *neoformans* Rim101, lacking the C-terminal 603 amino acids, in the *rim13Δ¸rim20Δ*, and *rim23Δ* mutant strains. In these “upstream” mutants, this constitutively active form of Rim101 (GFP-Rim101T) restores capsule formation, as well as growth on pH 8 and in the presence of 1.5 M NaCl (Fig 3A and 3B).

### ESCRT involvement in *C. neoformans* Rim pathway

In other fungi, the endosomal sorting complex required for transport (ESCRT) serves as a docking platform for Rim23, Rim20, and Rim13 proteins. Deletion of components of the ESCRT-I,-II, or-III complexes disrupts Rim101 activation [14,18,30–33]. In ascomycetes, the Rim pathway-dependent assembly of the ESCRT machinery is initiated by an interaction between the Rim8 arrestin-like protein and the ESCRT-I complex protein, Vps23. This recruits the ESCRT-II and then ESCRT-III complexes, eventually resulting in Rim101 proteolysis and activation [13,19,20].

Based on previous studies, there are several lines of evidence supporting a role for the ESCRT machinery in *C*. *neoformans* Rim101 activity. First, Hu et al., previously demonstrated that *vps23Δ* mutant phenotypes include sensitivity to pH 8 and 1.5M NaCl, along with displaying a capsule defect [34]. In addition, Chun and Madhani found that Vps25, a component of ESCRT-II, was required for several Rim101-dependent phenotypes [35]. More recently, Godinho et al demonstrated that the ESCRT-III component, Snf7, was required for capsule formation and full *RIM101* transcription [36]. Rim101 induces its own transcription, which may partially explain the reason that disruption of a Rim pathway component would decrease *RIM101* transcript levels [3]. Finally, both *C*. *neoformans* Rim23 and Rim20 contain predicted Snf7-interacting BRO1 domains (S1 Fig).

**Fig 4:**
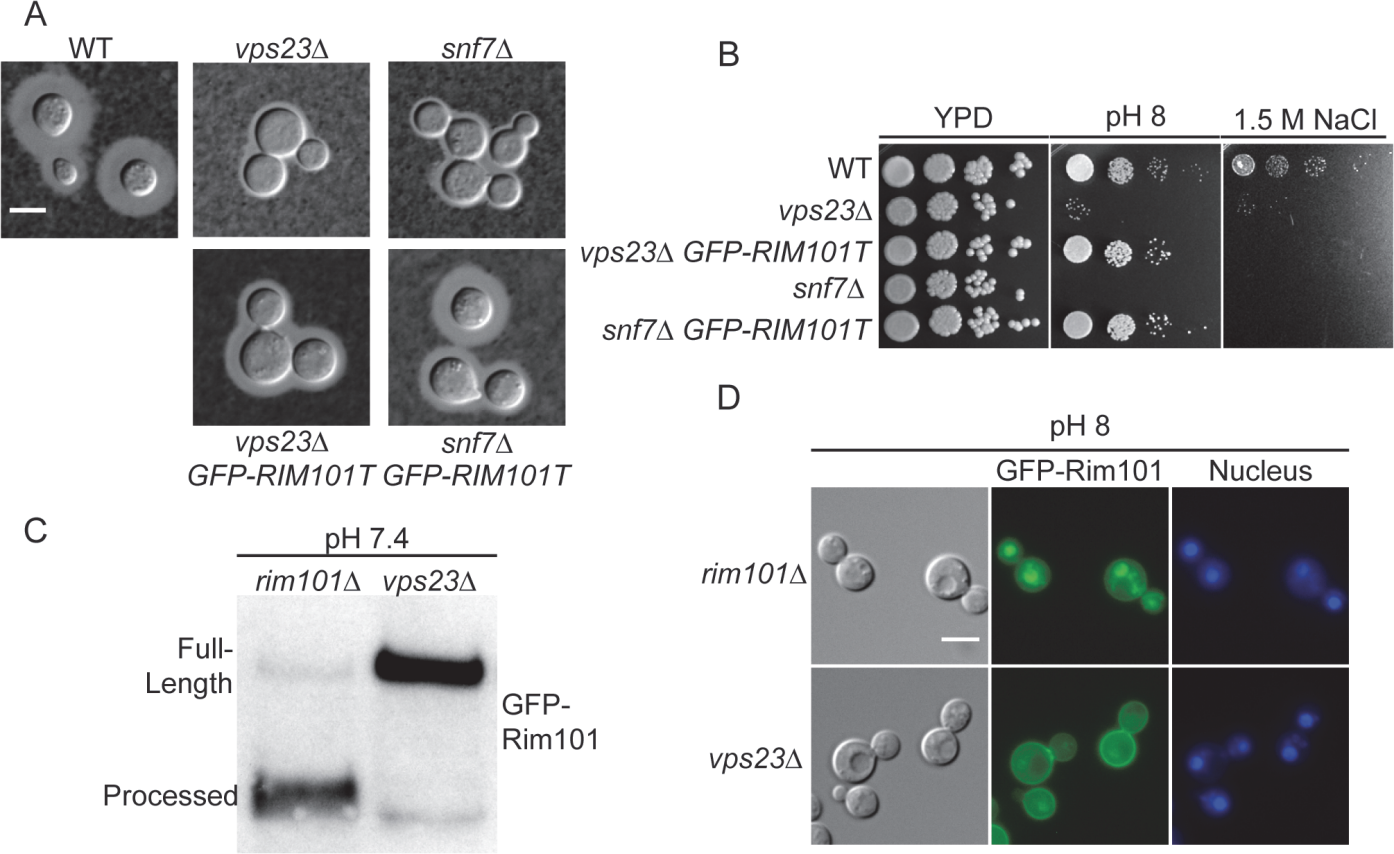
ESCRT complex proteins, Vps23 and Snf7, are required for Rim101 activation. (A) *snf7Δ* and *vps23Δ* capsule defects are partially rescued by *GFP-RIM101T* expression. Strains cultured for 24 hr in tissue culture media. India ink used to visualize capsule. (B) *GFP-RIM101T* expression rescues the *vps23Δ* and *snf7Δ* growth defects on pH 8 but not 1.5 M NaCl. (C) GFP-Rim101 was immunoprecipitated from the indicated strains after 5 hr incubation in YPD with 150mM HEPES at pH 7.4. (D) GFP-Rim101 (full-length) nuclear localization is disrupted in the *vps23Δ* mutant. Localization was assessed after culturing for 5 hr in SC with McIlvaine’s buffer at pH 8. Nuclei were stained with Hoechst 33342 live nuclear stain. Scale bar = 5 μm.

To directly determine whether the ESCRT complex is required for Rim101 activation in *C*. *neoformans*, we analyzed both the *vps23Δ* and *snf7Δ* mutants for defects in Rim pathway-associated phenotypes and Rim101 activation. Consistent with previously published data, both mutants displayed a marked capsule defect (Fig 4A). Both mutants were also sensitive to high salt and alkaline pH, similar to the *rim101Δ* mutant (Fig 4B) We also determined that GFP-Rim101 nuclear localization and proteolysis were disrupted in the *vps23Δ* mutation (Fig 4C and 4D). Similar to the *rim13Δ*, *rim20Δ*, *and rim23Δ* mutants, expression of the constitutively active, truncated GFP-Rim101T protein fully rescued the *vps23Δ* and *snf7Δ* mutant defects in growth at pH 8 and partially rescued their capsule defects (Fig 4A and 4B). Unlike the other Rim pathway mutants, GFP-Rim101T did not rescue the *vps23Δ* and *snf7Δ* growth defects on high salt concentrations. Therefore, both ESCRT-I and ESCRT-III components play conserved roles in *C*. *neoformans* Rim101 activation and function, although they have pleiotropic roles in regulating salt tolerance and complete capsule formation.

### Rim pathway membrane sensing complex components are not conserved in *C. neoformans*

In addition to the ESCRT machinery and the signaling components directly involved in Rim101 proteolytic activation, the classical ascomycete Rim pathway includes the integral membrane pH sensor Rim21/PalH, the integral membrane protein Rim9/PalI, and the arrestin-like Rim8/PalF. *S*. *cerevisiae* and *C*. *albicans* have an additional component of the Rim membrane sensing complex, Dfg16, which helps mediate Rim21/PalH localization and is also required for Rim pathway activation [16]. In ascomycetes, these plasma membrane Rim signaling components are identifiable based on highly conserved sequences and protein motifs. However, Rim21/PalH, Rim8/PalF, and Dfg16 orthologs could not be easily identified in *C*. *neoformans* using this approach. Similar searches did not identify predicted orthologs in any basidiomycete genome [26].

We also searched for predicted proteins with arrestin-like motifs that could be acting as Rim8 orthologs in *C*. *neoformans*. There were two predicted *C*. *neoformans* proteins (CNAG_02857, and CNAG_02341) that contained both the N- and C-terminal arrestin domains present in Rim8 proteins from other fungi. These were designated *ALI1* and *ALI2* (Arrestin Like 1 and 2). Disruption of these genes individually or in combination did not affect the Rim101-dependent phenotypes of capsule formation, growth at pH 8, or growth 1.5 M NaCl (S2 Fig) suggesting that these genes are not required for Rim101 activation.

We identified one predicted ortholog (CNAG_05654) of the Rim9 component of the sensing complex, which has a 24% sequence similarity to the *S*. *cerevisiae* Rim9 protein. We also identified 2 other genes (CNAG_02114 and CNAG_04953) predicted to encode proteins with homology to the SUR7/PalI domain, a defining feature of Rim9 proteins. Disruption of any of these potential *RIM9* orthologs, either separately or in combination in a triple mutant strain, did not affect Rim101-dependent phenotypes (S2 Fig).

### Identification of novel *C. neoformans* Rim pathway component

As we were unable to identify components of the *C*. *neoformans* Rim pathway membrane sensing complex using sequence comparisons or searches for conserved protein domains, we designed a forward genetics screen to specifically identify novel Rim101 activators. The defining characteristic of a Rim pathway mutant is that its mutant phenotypes are rescued by expression of the constitutively active form of Rim101. Therefore, we created a strain encoding the truncated *GFP-RIM101T* allele under the control of the galactose-inducible *GAL7* promoter, and we performed *Agrobacterium tumefaciens-*mediated random insertional mutagenesis in this strain. We then selected for mutants with *rim101Δ-*like phenotypes (sensitivity to alkaline pH and 1.5 M NaCl) in the presence of glucose (repressing conditions for GFP-Rim101T expression) and wild-type phenotypes on galactose-containing media (inducing conditions for GFP-Rim101T expression).

Using this random mutagenesis approach, we identified 40 mutants with galactose-suppressible alkaline and/or NaCl sensitivities. After identifying the site of each mutation, we recognized several disrupted genes among these mutants that were known to be required for Rim101 expression or activation. We identified one insertion in *HAPX*, which encodes a transcription factor required for full induction of *RIM101* expression [37]. In this case, galactose-induced expression of GFP-Rim101T likely rescued the defect in *RIM101* transcription, as opposed to a defect in Rim101 protein activation. We also identified an insertion in the *RIM13* gene encoding the Rim101 activating protease. These data demonstrate that this screening strategy was successful in identifying genes known to be required for Rim101 activity.

**Fig 5:**
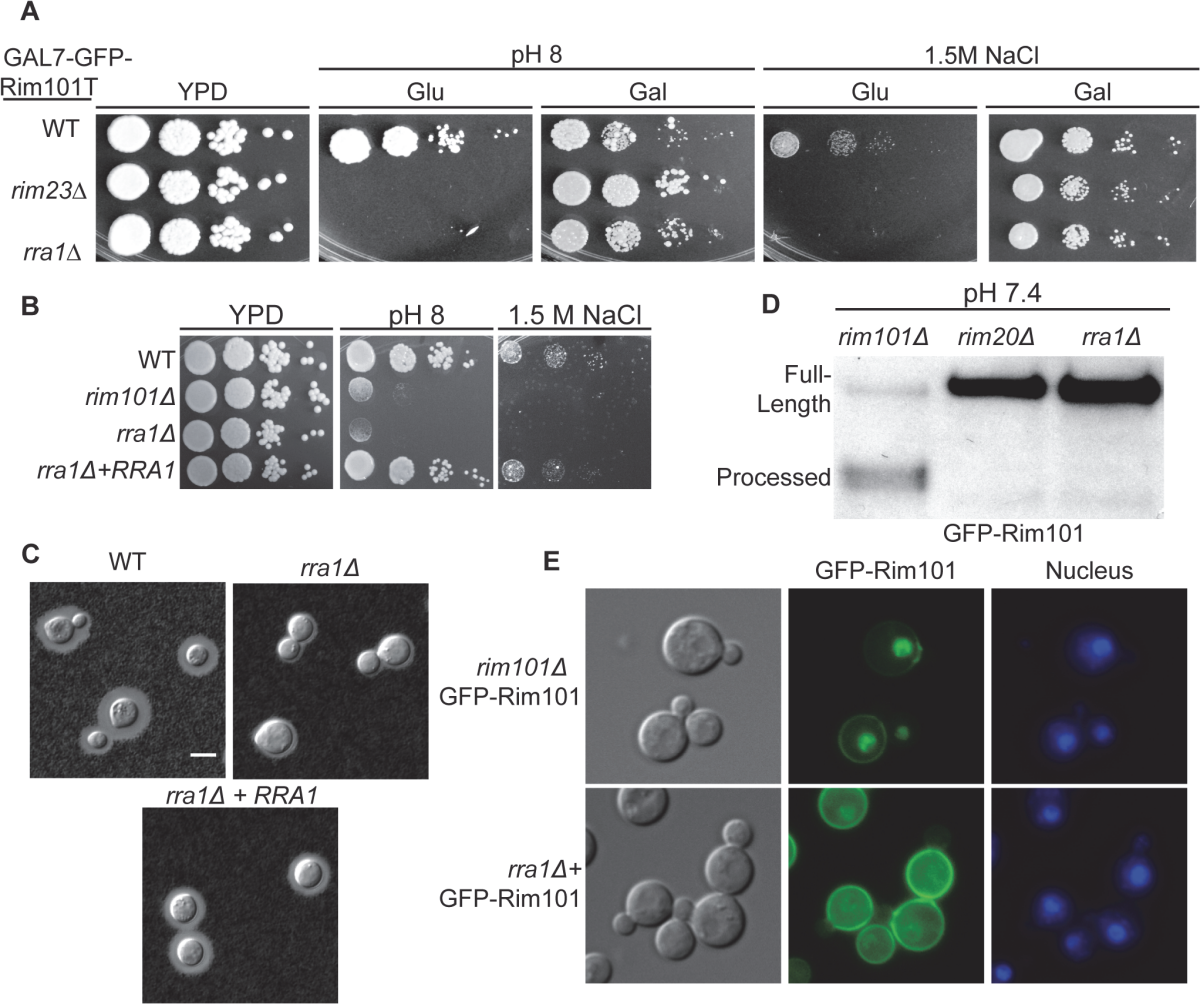
The *rra1Δ* mutant is phenotypically identical to other Rim pathway mutants. (A) *rra1Δ* insertional mutant has pH 8 and 1.5 M NaCl growth defects that are rescued by *GFP-RIM101T* expression. 10-fold serial dilutions were spotted onto YPD, YPD with 150 mM HEPES pH 8, and YPD with 1.5 M NaCl. (B) Independent *rra1Δ* mutant has a growth defect pH 8 and 1.5 M NaCl. (C) The *rra1Δ* strain has a capsule defect. Cells were cultured for 48 hr in CO_2_-independent media at 37^°^C to induce capsule. Capsule was visualized by India ink staining. Scale bar = 5 μm. (D) The *rra1Δ* mutation disrupts GFP-Rim101 proteolysis. GFP-Rim101 was immunoprecipitated from the indicated mutant strains after 5 hr incubation in YPD with 150 mM HEPES at pH 7.4. (E) GFP-Rim101 nuclear localization is disrupted in the *rra1Δ* mutant. GFP-Rim101 was assessed after 5 hr incubation in pH 8 SC McIlvaine’s buffer.

Among the additional mutants, we focused on a strain with an insertion in the CNAG_03488 locus due to its striking *rim101Δ-*like phenotypes on both 1.5 M NaCl and pH 8 (Fig 5A and 5B). Furthermore, these mutant phenotypes were completely rescued by the expression of GFP-Rim101T on galactose (Fig 5A). We confirmed these mutant phenotypes by independently disrupting the CNAG_03488 gene in the wild type H99 background (Fig 5B). In addition to pH 8 and 1.5M NaCl sensitivity, the CNAG_03488 mutant also displayed a *rim101Δ-*like capsule defect (Fig 5C). We complemented all mutant phenotypes by introduction of the wild type allele (Fig 5).

To determine if the protein encoded by the CNAG_03488 gene was a component of the Rim pathway, we assessed GFP-Rim101 localization and proteolysis in the CNAG_03488 mutant. Like the verified activators of Rim101, CNAG_03488 was required for both Rim101 cleavage and nuclear localization (Fig 5D and 5E). Based on these results, we concluded that, like *RIM13*, *RIM20*, *RIM23*, and the ESCRT complex, the novel protein encoded by CNAG_03488 is required for Rim101 activation. To reflect its role in the Rim pathway, we named this gene *RRA1* (Required for Rim101 Activation 1).

### Rra1 is conserved throughout Basidiomycota

**Fig 6:**
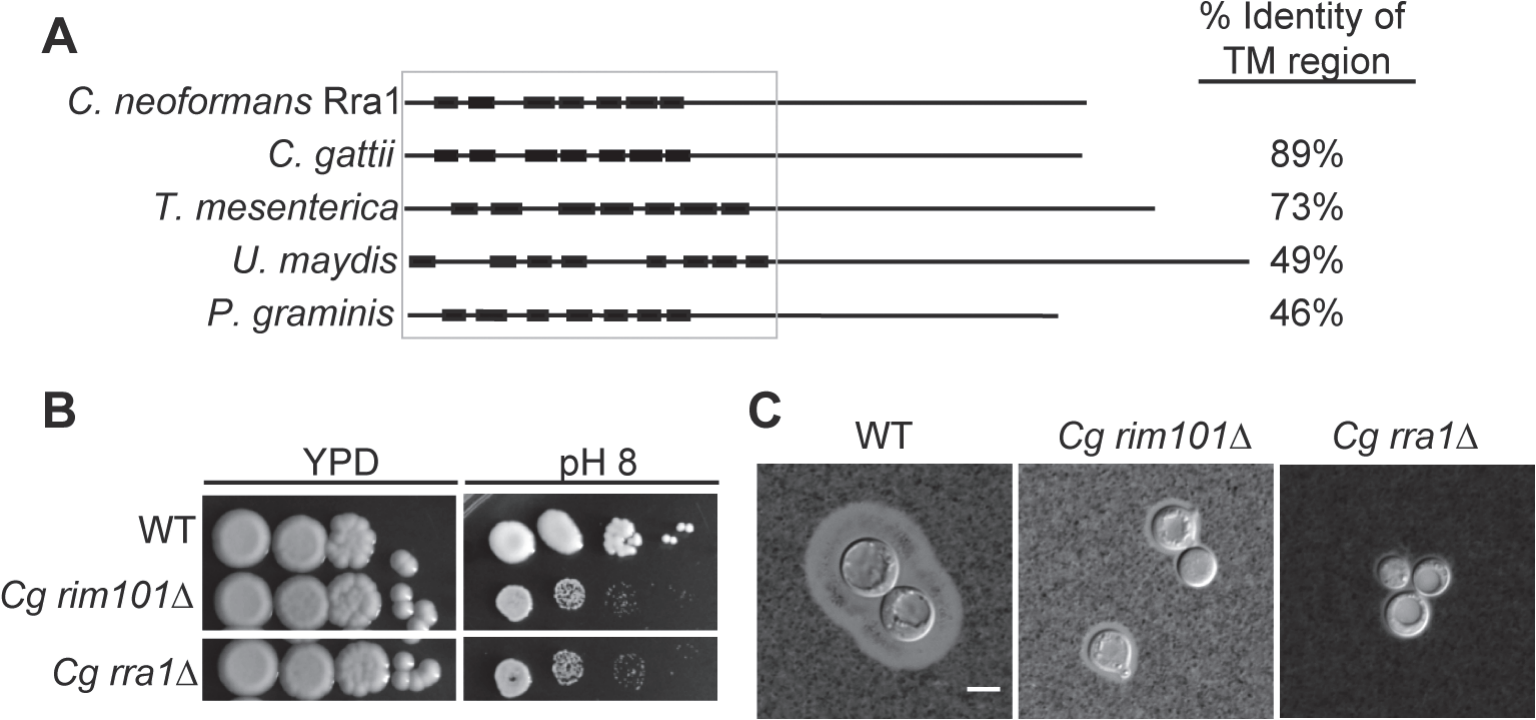
Rra1 is a membrane protein and is conserved through Basidiomycete fungi. (A) *C*. *neoformans* Rra1 and its predicted basidiomycete orthologs contain 7–8 predicted transmembrane domains (closed boxes). *C*. *neoformans* CNAG_03488 (Rra1), *C*. *gattii* CGB_G5320C, *Tremella mesenterica* TREME_69388, *Ustilago maydis* um00299, *Puccinia graminis* PGTG_03106 predicted protein sequences are modeled. The TM domains were predicted using The TMPred TM prediction server [38] The % identity to the transmembrane region of *C*. *neoformans* Rra1 was determined by comparing 1–290 of the predicted Rra1 amino acid sequence to each of the predicted orthologs. (B) *C*. *gattii rra1Δ* and *rim101Δ* mutants are sensitive to pH 8. (C) *Cg rra1Δ* and *rim101Δ* have a capsule defect. Capsules visualized with India Ink after 24 hr incubation in tissue culture media. Scale bar = 5 μm.

To determine whether Rra1 is a *C*. *neoformans-*specific or conserved component of the Rim pathway, we used the predicted Rra1 amino acid sequence to search for possible Rra1 orthologs in other fungal species. We only identified Rra1 homologs in other members of the basidiomycete phylum; however, these predicted proteins demonstrated a high degree of homology to Rra1 (Fig 6A and S3 Fig), even in distantly related members of this phylum such as *U*. *maydis* and *P*. *graminis*. We also demonstrated that Rra1 function in Rim pathway activation is functionally conserved in the divergent sibling species *Cryptococcus gattii*. Disruption of the *C*. *gattii RRA1* (CNBG_2126) ortholog resulted in alkaline sensitivity and a capsule defect identical to the *C*. *gattii rim101Δ* mutant (CNBG_4424) (Fig 6B and 6C). These results demonstrate that Rra1 orthologs function in Rim101-related processes in different basidiomycete species.

*C*. *neoformans* Rra1 and its predicted basidiomycete orthologs all contain 7–8 predicted transmembrane domains (Fig 6A and S3 Fig), suggesting that they may be structurally similar to the Rim21/PalH pH sensors, which also contain 7 transmembrane domains [21] However, Rra1 does not share significant amino acid sequence homology with Rim21/PalH proteins, and direct BLAST comparisons between Rra1 and *S*. *cerevisiae* Rim21 and *A*. *nidulans* PalH produced Expected values greater than 1.

### The Rim pathway does not require Gα protein activity

With 7 predicted transmembrane domains, Rra1 could also be structurally similar to GPCR proteins (G-protein Coupled Receptors). When activated, GPCRs interact with the Gα subunit of trimetric G proteins, leading to alteration in downstream signaling. *C*. *neoformans* encodes three characterized Gα proteins, Gpa1, Gpa2, and Gpa3 [39,40]. To begin to explore whether Rra1 is a GPCR and signaling through any of these Gα proteins, we analyzed the *gpa1Δ*, *gpa2Δ*, *and gpa3Δ* mutants for *rim101Δ-*like phenotypes. The *gpa1Δ* and *gpa3Δ* mutants grew like wild type on both pH 8 and 1.5 M NaCl, inconsistent with defective Rim pathway signaling. The *gpa2Δ* mutant was slightly sensitive to both pH 8 and 1.5 M NaCl, but these growth defects were not as severe as those displayed by a *rim101Δ* mutant or other Rim pathway mutants (S4 Fig). Moreover, Gpa2 plays a prominent role in *C*. *neoformans* mating, a cellular process that does not appear to be dependent on Rim signaling in this species [40]. Therefore, Gpa1, Gpa2, and Gpa3, were not required for Rim101 activation in *C*. *neoformans* and these proteins do not appear to be components of the *C*. *neoformans* Rim pathway.

### pH-regulated Rim23-GFP localization requires Rra1 and ESCRT

**Fig 7:**
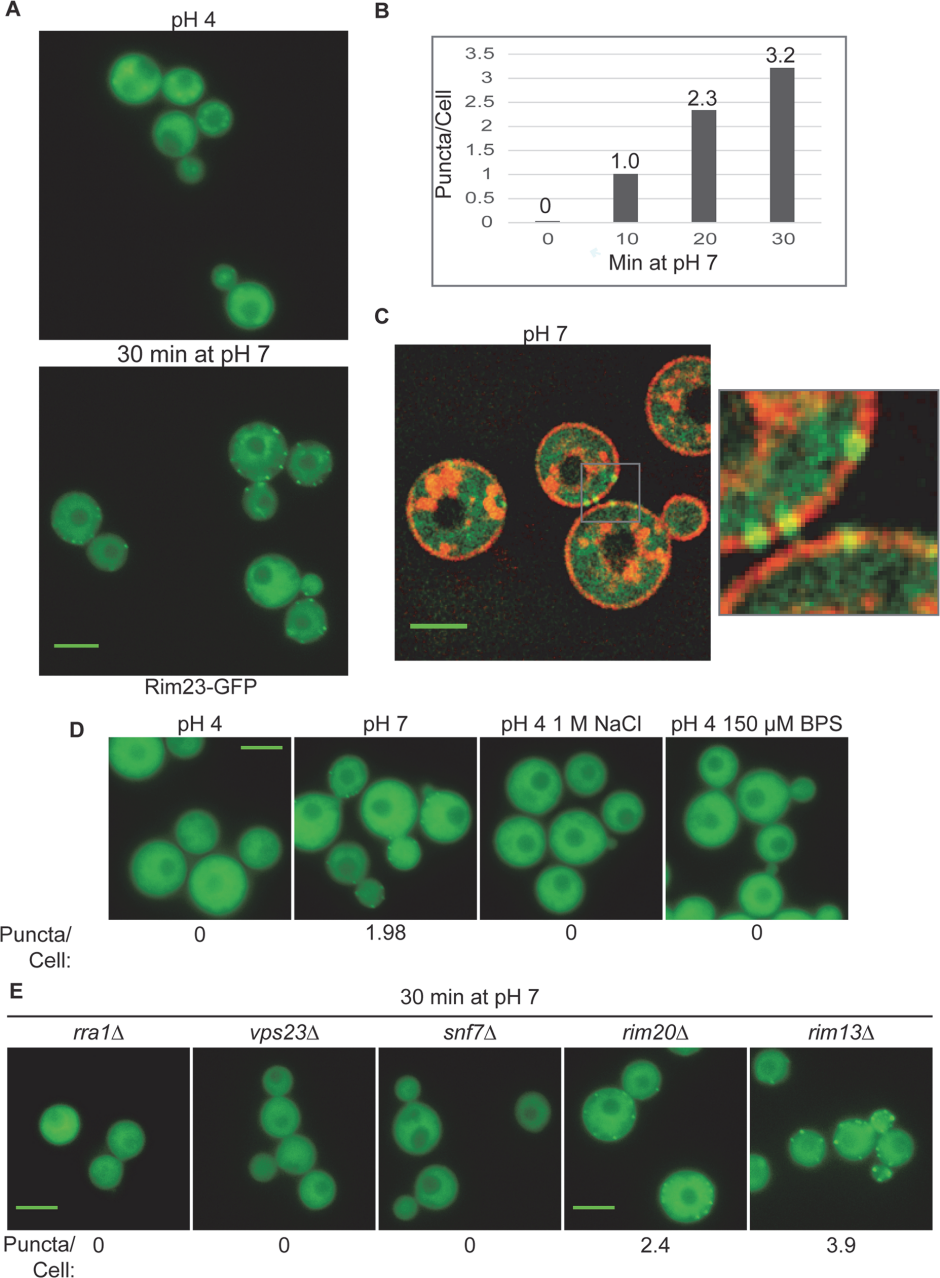
Rim23-GFP forms plasma membrane-associated puncta under neutral/alkaline pH conditions. (A) Rim23-GFP was visualized at pH 4 SC McIlvaine’s buffer and after 30 min after shift to pH 7 SC McIlvaine’s buffer. (B) The number of Rim23-GFP puncta increase with time after shifting from pH 4 to pH 7. Quantification Rim23-GFP puncta/cell at pH 4 (0 min) and after 10, 20, 30 min incubation at pH 7. (C) Rim23-GFP puncta are closely associated with the plasma membrane. Cells were stained with FM-464 after 1 hr incubation at pH 7. (D) 1 M NaCl and 150 μM BPS does not induce Rim23-GFP puncta formation. Cells images after 30 min incubation in indicated culture media. (E) Rim23-GFP puncta formation was disrupted by *rra1Δ*, *vps23Δ*, and *snf7Δ* mutants. Cells were imaged after 30 min incubation at pH 7. The number of puncta/cell was quantified for 62–100 cells/strain. All strains in this figure were cultured in SC McIlvaine’s buffer media. All scale bars = 5 μm.

When activated, the ascomycete Rim21/PalH sensor recruits downstream Rim pathway components to punctate structures at the plasma membrane [12,14,19,21]. The order in which these components localize to this complex was used to establish the sequence of Rim/Pal protein activation (Fig 1). To determine if similar pH-regulated localization changes occur in *C*. *neoformans*, we expressed a Rim23-GFP fusion protein, regulated by the endogenous *RIM23* promoter, in a *rim23Δ* mutant. This fusion protein was functional, fully rescuing the *rim23Δ* defects in pH 8 growth and capsule formation, and partially rescuing the 1.5 M NaCl growth defect. When incubated at pH 4, Rim23-GFP localized diffusely throughout the cell (Fig 7A). After shifting the cells to pH 7, Rim23-GFP migrates to distinct, cell surface-associated puncta. The number of puncta/cell increased from 1 to 3.2 between 10 min and 30 min of incubation at neutral pH (Fig 7B). These puncta appear to be on the plasma membrane or very close to the plasma membrane, co-localizing with non-endocytosed FM4-64 staining of the plasma membranes (Fig 7C). These punctate structures formed specifically in response to elevated pH, and not to other stress conditions associated with Rim101 function, such as high salt or low iron: the GFP-Rim23-containing puncta were not observed in cells shifted to pH 4+1 M NaCl, or pH 4+150 μM BPS (Fig 7D). Together, these data indicate that Rim23 cell surface puncta are specifically induced in response to pH signals, consistent with a role for neutral/alkaline pH in inducing Rim101 activation.

We next determined which components of the Rim pathway were required for the observed Rim23-GFP plasma membrane localization. Neither Rim20 nor Rim13, other members of the Rim proteolysis complex, were required for Rim23-GFP cell surface puncta formation. However, pH-driven Rim23 localization to surface punctate structures did not occur in the *rra1Δ*, *vps23Δ* and *snf7Δ* mutants (Fig 7E). Therefore, Rim20 and Rim13 are not required for Rim23 localization, while both the ESCRT pathway proteins and Rra1 are functionally upstream of Rim23-puncta formation.

### Rim pathway mutants induce an increased inflammatory response during infection

**Fig 8:**
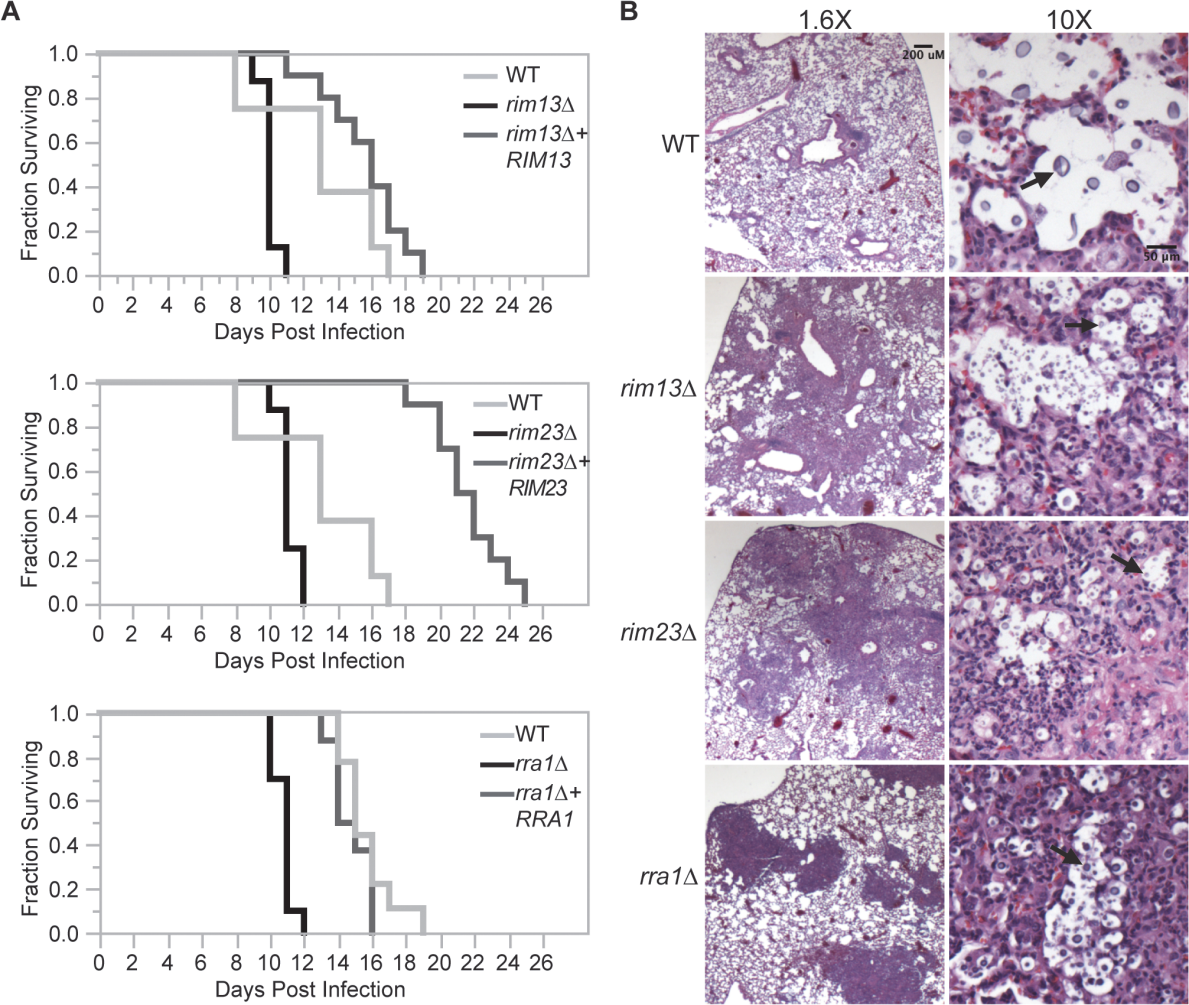
Effects of Rim pathway mutants on virulence. (A) *rim13Δ*, *rim23Δ*, and *rra1Δ* mutants are hypervirulent in a murine model of cryptococcosis. 8–10 A/Jcr female mice were intranasally inoculated with 1X10^5^ cryptococcal cells and monitored daily for survival. (B) Histopathological analysis revealed increased inflammatory cell infiltration in Rim-pathway mutants. Infected A/Jcr mouse lungs were harvested on day 7 post inoculation and H & E stain. Black arrows mark *C*. *neoformans* cells.

During infection, *C*. *neoformans* Rim101 regulates the expression of several genes involved in cell wall synthesis and maintenance by directly binding to elements in their promoters [3,11]. In the absence of Rim101-directed cell wall maintenance, the *rim101Δ* strain induces a hyper-inflammatory response in the lungs of infected mice. In this way, infection with this attenuated strain results in the paradoxical death of the host. We previously demonstrated that Rim101 activity is also regulated by the PKA/cAMP pathway, and *pka1Δ* mutant displays cell wall changes similar to the *rim101Δ* mutant [3,11]. As Rim101 activation is regulated by both the cAMP/PKA pathway and the Rim pathway, we examined whether mutations in other components of the Rim pathway were sufficient to alter virulence in a murine inhalation model of cryptococcosis. For each strain, 8–10 A/J mice were intranasally inoculated with 5x10^4^ *C*. *neoformans* cells. Mice were monitored daily and sacrificed when they displayed symptoms of disease consistent with imminent death. In each experiment, all mice infected with the wild type died between 18–20 days. In contrast to the mice infected with the wild type and reconstituted strains, all mice infected with the *rim13Δ*, *rim23Δ*, or *rra1Δ* mutant cells succumbed to the infection by day 12 (Fig 8A). This statistically significant decrease in survival (p>0.001) was consistent with the mortality of mice infected with a *rim101Δ* mutant [9,11]. Both the *rim13Δ+RIM13* and *rra1Δ+RRA1* reconstituted strains resulted in animal mortality at a rate very similar to WT. The *rim23Δ+RIM23* reconstituted strain displayed a mild delay in virulence, which we subsequently found was likely due to a temperature sensitive phenotype specific only to this isolate. An independent *rim23Δ+RIM23* isolate without a temperature-sensitive phenotype was used for the rest of these experiments.

Like the *rim101Δ* mutant, the *rim13Δ*, *rim23Δ*, and *rra1Δ* mutants induced an increased infiltration of inflammatory cells in the lungs of infected mice. Histologic examination of infected mouse lungs 7 days after infection revealed that, unlike the lungs infected with wild type, those infected with Rim pathway mutants were characterized by diffuse regions of intense inflammatory cell infiltration (Fig 8B). This type of inflammation is consistently observed in mice infected with the *rim101Δ* mutant strain [11]. These results suggest that the immune system recognizes other Rim pathway mutants in a similar manner to a *rim101Δ* mutant.

The histopathological images also allowed us to analyze size and morphology of the *C*. *neoformans* cells. In the wild type infected lungs, there were numerous titan-sized cells [41,42]. In contrast, the *rim13Δ*, *rim23Δ*, and *rra1Δ* mutant cells were noticeably smaller in size (Fig 8B). Therefore, like *rim101Δ*, these mutants also have a defect in titan cell formation in vivo [43]. Together, these results are consistent with a model in which Rim13, Rim23, and Rra1 act as mediators of Rim101 activation in the context of infection.

## Discussion

**Fig 9:**
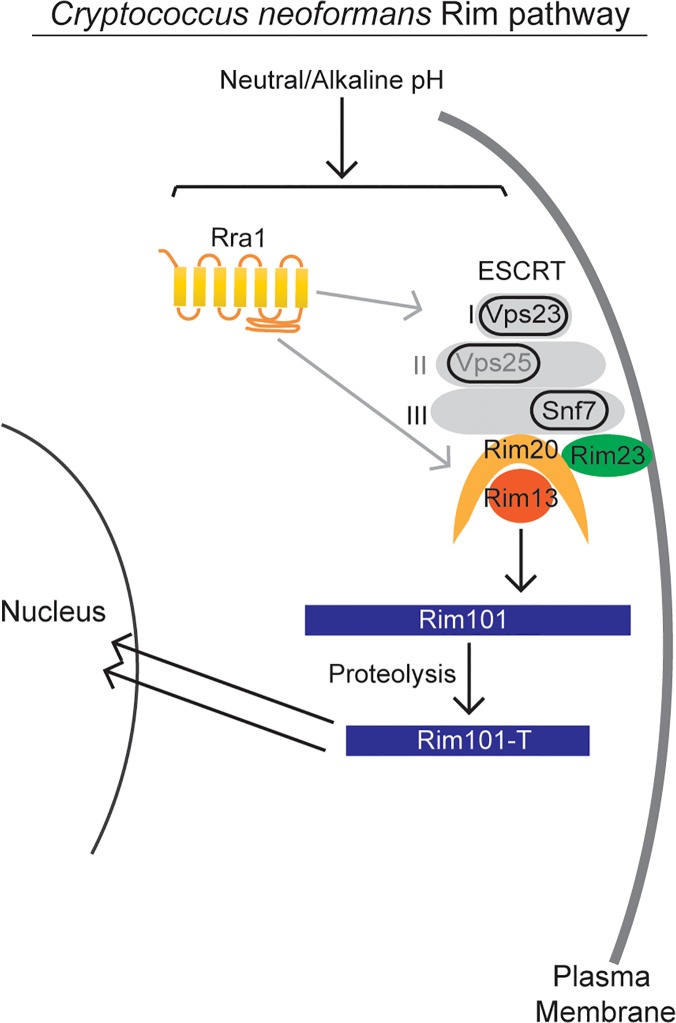
Model of *C*. *neoformans* Rim pathway. Our data supports a model in which the Rim pathway is activated by neutral/alkaline pH. In this pathway, the newly discovered Rra1 membrane protein and the ESCRT complex, including Vps23 (ESCRT-I) likely Vps25 (ESCRT-II) and Snf7 (ESCRT-III), induce Rim23 puncta formation and activate the Rim101-proteolysis complex. The Rim13 protease cleaves the Rim101 transcription factor, inducing its nuclear localization. Active Rim101 then transcriptional changes necessary for adapting to alkaline pH.

In this study, we identified both conserved and novel components of the *C*. *neoformans* Rim pathway. Based on our results as well as investigation of this pathway in other fungi, we propose the model in Fig 9 for Rim101 activation in *C*. *neoformans*. In response to neutral/alkaline pH, both the newly discovered Rra1 membrane protein and the ESCRT—I,-II,-III complex proteins control Rim23 relocation to punctate structures on the plasma membrane. In addition to Rim23, the other components of the Rim101 proteolysis complex are also functionally conserved in *C*. *neoformans*. Proteolysis enhances Rim101 nuclear localization, where it acts as both a transcriptional activator and repressor of a large group of genes, some of which are necessary for adaption to neutral/alkaline pH [3,11].

While the *C*. *neoformans* Rim pathway is required for responding to several stressful conditions, our results suggest that it is specifically activated by increased pH. We found that neither iron limitation nor high NaCl concentrations could alone induce Rim101 processing. In contrast, increasing pH resulted in a dose-dependent increase in Rim101 proteolytic processing and nuclear localization. The marginally increased intensity of GFP-Rim101 nuclear staining under iron limitation was likely due to increased levels of full-length Rim101; which may possess some intrinsic signaling activity. Rim101 transcription is activated by iron-responsive transcription factors, such as HapX, which themselves are activated by this low-iron growth condition [37] The exact mechanism of Rim pathway pH sensing is still largely unknown in any system, although recent research in *S*. *cerevisiae* Rim signaling suggests that altered membrane polarity serves as at least one of the pathway activating signals [12,44].

The ascomycete Rim101/PalC proteolysis complex consists of Rim23/PalC, Rim20/PalA, and Rim13/PalB. Upon pathway activation, these proteins are recruited by the ESCRT complex to plasma membrane puncta [12,14,19]. We demonstrate here demonstrate that the Rim101 proteolysis complex is functionally conserved in *C*. *neoformans* and is likely assembled and localized similarly to the ascomycete Rim101/PacC proteolysis complex. In addition to the required role of Rim23, Rim20 and Rim13 in Rim101 activation, we demonstrated that Rim23 is recruited to plasma membrane puncta when the pathway is activated. Rim23 is likely recruited to these punctate structures through an interaction with the assembled ESCRT machinery. The majority of the Rim23 protein sequence consists of the conserved Snf7-interacting domain, which likely facilitates an ESCRT-III/Rim23 interaction. Further supporting this model, both Vps23 (ESCRT-I) and Snf7 (ESCRT-III) were required for Rim23 puncta formation. The same Snf7-interacting domain is also present in the Rim20 amino acid sequence, indicating that, like in ascomycetes, both Rim23 and Rim20 interact with the assembled ESCRT-III complex in *C*. *neoformans*. [14,19,21]. Taken together, our results suggest that the *C*. *neoformans* Rim101 proteolysis complex is recruited to the plasma membrane after Rim pathway activation and organized in a similar manner to the ascomycete Rim101 proteolysis complex.

The role of the *C*. *neoformans* ESCRT complex in Rim101 activation has been suggested by several studies on ESCRT components [34,45,46]. We demonstrated that at least two documented ESCRT-mutant phenotypes, alkaline pH sensitivity and a capsule formation defect, result from failed Rim101-activation. This suggests that other ESCRT-dependent processes, such as heme acquisition [34], may also be dependent on the Rim pathway. However, we also observed that the ESCRT complex regulates multiple processes independent of Rim101 activation. First, the *vps23Δ* and *snf7Δ* mutants displayed NaCl sensitivity that was more severe than other Rim pathway mutants, and this was not rescued by expression of the constitutively active Rim101T. Furthermore, constitutively active Rim101T expression only partially rescued the *vps23Δ* and *snf7Δ* capsule defects. Perhaps the most striking difference between the ESCRT mutants and other Rim pathway mutants is their differing roles in pathogenesis. Unlike the Rim pathway mutants, which are hypervirulent in a murine inhalation infection model, both *snf7Δ* and *vps23Δ* mutants are avirulent [34,36]. These more severe ESCRT mutant phenotypes reflect the multiple roles the ESCRT machinery serves in *C*. *neoformans* biology and pathogenesis.

Prior to this study, the only identified basidiomycete Rim pathway components were identified based on sequence similarity to components of the ascomycete Rim/Pal pathway [25,26]. This strategy failed to identify the most upstream Rim pathway components in both *U*. *maydis* and *C*. *neoformans* or other members of this phylum. In this study, we performed a forward genetics screen and identified a novel component of the *C*. *neoformans* Rim pathway. This novel protein, Rra1, is functionally conserved in at least one related basidiomycete, *C*. *gattii*, and is conserved at a sequence level in more distantly related basidiomycetes. Rra1 is required for all Rim101-dependent phenotypes and is functionally upstream of the pH-regulated subcellular localization of the Rim101 proteolysis complex protein, Rim23. Unlike the ESCRT complex components, disruption of *RRA1* did not lead to additional, non-Rim101 dependent phenotypes, suggesting that Rra1 functions specifically in Rim pathway activation.

While the exact function of Rra1 is unknown, it has two characteristics that suggest that it may function as a Rim pathway pH sensor. Like the ascomycete Rim21/PalH pH sensor, Rra1 was required for neutral/alkaline pH induced Rim23 puncta formation [14]. In addition, Rra1, in *C*. *neoformans* and other basidiomycetes, is predicted to contain 7 transmembrane domains. However, Rra1 does not share significant amino acid sequence homology with any Rim21/PalH protein. Since the exact Rim21/PalH amino acids responsible for sensing pH are unknown, we are as yet unable to determine if Rra1 also contains similar pH sensing domains. Rra1 may also sense pH through histidine or paired glutamic acid/aspartic acid residues that are common in other pH sensing GPCRs or ion transporting proteins, and there are several of these amino acids that appear within the basidiomycete Rra1 amino acid sequence [47–50]. While the predicted Rra1 secondary structure suggests a functional similarity to Rim21/PalH, it is also possible that Rra1 serves a different purpose in Rim pathway activation. Like Dfg16 in *S*. *cerevisiae*, Rra1 may serve a supportive role in the proper localization of the Rim pathway pH sensor [12,16,19]. Rra1 may also function in a different cellular location, such as the vacuolar membrane or ER, which are known sites of pH-sensing and pH homeostasis [51,52]. Further studies on this novel protein will significantly advance our understanding of how *C*. *neofromans*, and other basidiomycetes sense pH.

Other components of the *C*. *neoformans* Rim membrane sensing complex remain elusive. None of the three potential Rim9/PalI orthologs nor the two candidate Rim8/PalF arrestin-like proteins were required for Rim pathway activation. Given the Rra1 structural similarity to GPCRs, we also examined whether the Rim pathway could signal through a canonical Gα protein, but none of the three known Gα proteins were required for Rim pathway activation. More extensive genetic screens will likely identify additional components for the *C*. *neoformans* Rim pathway, including cell surface chaperones and linking proteins.

The role of the Rim pathway in *C*. *neoformans* pathogenesis is complex. First, this pathway is required for *C*. *neoformans* survival in the host and *rim101Δ* mutants are rapidly and efficiently cleared in animal models of infection. Mechanistically, we have demonstrated that Rim101 is required for survival in the face of specific conditions found in micro-niches of the host; alkaline pH, iron deprivation, salt stress. Paradoxically, while these attenuated strains are poorly viable, they simultaneously induce an overly exuberant immune response. This is likely due to failed organization of the cell wall-masking phenotype and exposure of highly immunogenic epitopes. Interestingly, in human cases of cryptococcal meningitis, transcriptional studies of the pathogens at the site of infection demonstrate that *RIM101* transcripts are among the most highly induced during infection [53]. These previous studies suggested that Rim101-regulated cell wall changes in *C*. *neoformans* may be induced by the cAMP/Pka1 pathway. Here, we show that other elements of the Rim pathway are also required for Rim101-mediated interactions with the host.

In this study, we have provided the most thorough investigation of the Rim pathway in any basidiomycete fungus. Together, these data demonstrate the importance of microbial adaptation to environmental and host-specific signals for pathogen survival. Additionally, they further enrich our understandings of pathogen adaptation and host immune recovery to minimize host damage during infections.

## Materials and Methods

### Strains, media, and growth conditions

Gene loci analyzed in this study are shown in Table 1.The strains used in this study are shown in Table 2. The *C*. *neoformans* H99 Matα genetic background was used to create each mutant and fluorescent strain [54]. Unless otherwise stated, cells were grown in YPD (1% yeast extract, 2% peptone, 2% dextrose) [55]. To create the *eGFP-RIM101* strains (KS88-2, KS87-2, and KS118) each mutant strain was crossed with KN99 Mat**a** on MS mating media [56]. Spores were isolated by microdissection and recombinant spores were identified by PCR and nourseothricin (*NAT*) or neomycin (*NEO*) resistance. The resulting Mat**a** mutant strains were crossed with *rim101Δ* + *GFP-RIM101* (TOC105) and recombinant spores were isolated by microdissection. The *rim90Δ rim91Δ rim92Δ* (KS136) strain was created by first creating *rim91Δ rim92Δ* MAT**a** KS117 and crossing it with the KS63 strain. The *GAL7-RIM101 70T* strains were created by crossing *rim23Δ* + *GAL7-RIM101 70T* (KS159) with KN99 Mat**a** and isolating recombinant progeny. The KS161 strain was identified by PCR and phenotype.

**Table 1:** Gene loci analyzed in this study.

Genes	CNAG number	GeneBank
*RIM13*	CNAG_05601	AFR99029.2
*RIM20*	CNAG_03582	AFR96803.1
*RIM23*	CNAG_02205	AFR95615.1
*RRA1*	CNAG_03488	AFR96713.2
*RIM90*	CNAG_05654	AFR99085.1
*RIM91*	CNAG_02114	AFR94219.1
*RIM92*	CNAG_04953	AFR94219.1
*ALI1*	CNAG_02857	AFR93834.1
*ALI2*	CNAG_02341	AFR95488.1
*CgRIM101*	CNGB_4424	
*CgRRA1*	CNGB_2126	

**Table 2:** Strain list.

Strains	Genotype	Source
H99	*MAT*α	[57]
KN99	*MAT* ***a***	[58]
KS33	*rim13Δ*::*NEO MATα*	this study
KS110	*rim13Δ*::*NEO MAT* ***a***	this study
TOC66	*rim13Δ*::*NEO + RIM13 + pCH233 (NAT) MATα*	this study
KS88-2	*rim13Δ*::*NEO + eGFP-RIM101 pCH233 (NAT) MATα*	this study
KS140	*rim13Δ*::*NEO +GFP-RIM101T +* pCH233 (*NAT*) MATα	this study
KS94	*rim23Δ*::*NEO MATα*	this study
KS82-2	*rim23Δ*::*NEO MAT* ***a***	this study
KS81-2	*rim23Δ*::*NEO + RIM23 + pCH233 (NAT) MATα*	this study
KS87-2	*rim23Δ*::*NEO + eGFP-RIM101 +* pCH233 *(NAT) MATα*	this study
KS141	*rim23Δ*::*NEO +GFP-RIM101T +* pCH233 (*NAT*) *MATα*	this study
KS159	*rim23Δ*::*NEO +* pTO22 (*GAL7-GFP-RIM101T NAT) MATα*	this study
TOC17	*rim20Δ*::*NAT MATα*	[8]
KS118-2	*rim20Δ*::*NAT + eGFP-RIM101* pJAF (*NEO*) MATα	this study
KS77-2	*rim20Δ*::*NAT +GFP-RIM101T +* pJAF (*NEO*) *MATα*	this study
TOC2	*rim101Δ*::*NAT MATα*	[8]
TOC105	*rim101Δ*::*NAT + eGFP-RIM101* MATα	[2]
KS208	*rim101Δ*::*NAT + eGFP-RIM101 MAT* ***a***	this study
TOC65	*rim101Δ*::*NAT* + *GFP-RIM101T +* pJAF (*NEO*) *MATα*	this study
*vps23-9*	*vps23Δ*::*NEO MATα*	[20]
KS211	*vps23Δ*::*NEO + eGFP-RIM101 +* pCH233 *(NAT) MATα*	this study
KS75-2	*vps23Δ*::*NEO +GFP-RIM101T +* pCH233 (*NAT*) MATα	this study
*snf7Δ*	*snf7Δ*::*NEO*	[36]
KS151	*snf7Δ*::*NEO +GFP-RIM101T +* pCH233 (*NAT*) MATα	this study
KS63	*rim90Δ*::*NEO MATα*	this study
KS57	*rim91Δ*::*NEO MATα*	this study
KS53	*rim92Δ*::*NEO MATα*	this study
KS117	*rim91Δ*::*NEO rim92Δ*::*NEO MAT* ***a***	this study
KS136	*rim90Δ*::*NEO rim91Δ*::*NEO rim92Δ*::*NEO MATα*	this study
KS118	*ali1Δ*::*NEO MATα*	this study
KS94-2	*ali2Δ*::*NAT MATα*	this study
KS97-2	*ali1Δ*::*NEO ali2Δ*::*NAT MATα*	this study
KS161	*H99 +* pTO22 (*GAL7-GFP-RIM101T NAT) MATα*	this study
KS216	*AGRO MUTANT rra1Δ*::*NEO +* (GAL7-GFP-RIM101T NAT) MATα	this study
KS183	*rra1Δ*::*NEO MATα*	this study
KS202	*rra1Δ*::*NEO +* pKS38 (*RRA1 NAT*) *MATα*	this study
KS185	*rra1Δ*::*NEO + eGFP-RIM101 MATα*	this study
KS214	*rra1Δ*::*NEO +* pKS34 (p*HIS3-GFP-RRA1 NAT*) *MATα*	this study
KS289	*rim23Δ*::*NEO* + *GFP-RIM23 + NAT MATα*	this study
KS290	*rim23Δ*::*NEO* + *GFP-RIM23 + NAT MAT* ***a***	this study
KS292	*snf7Δ*::*NEO rim23Δ*::*NEO* + *GFP-RIM23 +* pCH233 (*NAT)*	this study
KS296	*vps23Δ*::*NEO rim23Δ*::*NEO* + *GFP-RIM23 +* pCH233 (*NAT)*	this study
KS298	*rim13Δ*::*NEO rim23Δ*::*NEO* + *GFP-RIM23 +* pCH233 (*NAT)*	this study
KS299	*rim20Δ*::*NEO rim23Δ*::*NEO* + *GFP-RIM23 +* pCH233 (*NAT)*	this study
KS301	*rra1Δ*::*NEO rim23Δ*::*NEO* + *GFP-RIM23 +* pCH233 (*NAT)*	this study
YSB25	*gpa2Δ*::*NEO*	[40]
YPH105	*gps3Δ*::*NEO*	[40]
YSB83	*gpa1Δ*::*NAT*	[59]
R265	*C*. *gattii*	[60]
KS260	*C*. *gattii rra1Δ*::*NEO*	this study
KS261	*C*. *gattii rim101*::*NEO*	this study

YP-Gal media contained 1% yeast extract, 2% peptone, and 3% galactose. The pH 4 and pH 8 media was made by adding 150 mM HEPES buffer to YPD or YP-Gal liquid media, adjusting the pH with concentrated HCl (for pH 4) or NaOH (for pH 8), prior to autoclaving. The NaCl plates were made by adding NaCl to YPD to a concentration of 1.5 M. To induce capsule, strains were incubated for 48 hr in CO_2_-independent medium (Gibco) with 150 rpm shaking at 37^°^C.

To analyze GFP-Rim101 localization, strains were grown overnight (~18 hr) at 30^°^C with 150 rpm shaking. Cells were then pelleted and resuspended in either pH 4 or pH 8 Synthetic Complete media buffered with McIlvaine’s buffer. Strains were shaken at 150 rpm, 30^°^C for 5 hr

### Molecular biology

The primers used to create each mutant and fluorescent strain are listed in Table 3.

**Table 3:** Primers.

Primer name	5'-3' Primer sequences
AA1792	GTCATAGCTGTTTCCTGGGGCGGATGATGCAGAGTTA
AA1793	ACTGGCCGTCGTTTTACTAACAAAGGTAACCGTCGGT
AA1794	ACCGACGGTTACCTTTGTTAGTAAAACGACGGCCAGT
AA1795	AAGTCAGCGGTCTTGAGGAA
AA1796	TAAGGGCTAAAGTCGGAGCA
AA1797	TAACTCTGCATCATCCGCCCCAGGAAACAGCTATGAC
AA1812	ATCTTGCCATTGATGATAG
AA1813	GTCGGAAGATTAAAAAGTG
AA3226	GGATTGGTCTAGGGCCTCTT
AA3227	GTCATAGCTGTTTCCTGTATGTATAATGATTATATCTG
AA3228	CAGATATAATCATTATACATACAGGAAACAGCTATGAC
AA3229	CGTATATCATATTCAACTTTCTCGTTTTCCCAGTCACGAC
AA3230	GTCGTGACTGGGAAAACGAGAAAGTTGAATATGATATACG
AA3231	ATTTAGCCCCGTCGTCTTCT
AA3241	CCGATGTAGTGGCCAAGTCT
AA3242	TGGACATACCAGACGATCCA
AA3254	GAGGACTACTTGGGCGTCAA
AA3255	GTCATAGCTGTTTCCTGCTGTCGGACCGTGTTTATCG
AA3256	CGATAAACACGGTCCGACAGCAGGAAACAGCTATGAC
AA3257	ATATTATAAGTTAGAGGTTAGGTTTTCCCAGTCACGAC
AA3258	GTCGTGACTGGGAAAACCTAACCTCTAACTTATAATAT
AA3259	GGACGGGAGTGTAATGAGGA
AA3260	TGTGCATTCTGCATGGTTTT
AA3261	GTCATAGCTGTTTCCTGGTTTTATAGTTCCGAAGTTGAC
AA3262	GTCAACTTCGGAACTATAAAACCAGGAAACAGCTATGAC
AA3263	GGATGGAATTATAGAATGGCGTTTTCCCAGTCACGAC
AA3264	GTCGTGACTGGGAAAACGCCATTCTATAATTCCATCC
AA3265	CTTCGCCCTTTGATCTTGAG
AA3267	AAGATGTGATCGCGTGAATG
AA3268	GTCATAGCTGTTTCCTGGATGGCAGTTTAGTTGTGAG
AA3269	CTCACAACTAAACTGCCATCCAGGAAACAGCTATGAC
AA3270	GATGAAGATGGCAAAATATATTGTTTTCCCAGTCACGAC
AA3271	GTCGTGACTGGGAAAACAATATATTTTGCCATCTTCATC
AA3272	TGAAGAAAGGGGAGGTGATG
AA3292	GCTTTTGATGACCCTGTCGT
AA3293	CCAAAGACGTGTGATTGTGG
AA3294	TGGTGATCCATGCTTGTTGT
AA3295	AATTTATCCGGGAGGAATCG
AA3296	ATTTCTTACGGCCGGAACTT
AA3361	GACTGGGCCTATGTTGAGGA
AA3362	GTCATAGCTGTTTCCTGCGTGAGGTGTAGGGAAGGAGCAG
AA3363	CTGCTCCTTCCCTACACCTCACGCAGGAAACAGCTATGAC
AA3364	GCAAAATAAAAAGAATGTATCAAGTTTTCCCAGTCACGAC
AA3365	GTCGTGACTGGGAAAACTTGATACATTCTTTTTATTTTGC
AA3366	ACGAATAATAGGGGGCATCC
AA3401	ACGAATAATAGGGGGCATCC
AA3402	ACATCGCATCTCGAGGTTTC
AA3505	CTGAGCGGTGTCCTTTTCTC
AA3506	GTCATAGCTGTTTCCTGGGTGTGGGTGTGGTTGTCGTGGT
AA3507	ACCACGACAACCACACCCACACCCAGGAAACAGCTATGAC
AA3508	GTATATCTAGATTGAACAACTAAGTTTTCCCAGTCACGAC
AA3509	GTCGTGACTGGGAAAACTTAGTTGTTCAATCTAGATATAC
AA3510	TTTCAGTTCCGAGGTGCTCT
AA3672	GATTCGCACCATTGGTCTTT
AA3673	TAACGCGGAGCTCTGATCTT
AA4011	CTCCCTCCACCAGATACCAA
AA4012	CTGCCACAAAGTTGAACGTC
AA3970	ACCACCACCATCCTAACCAG
AA3971	ACGAGGAAGAAGGGTAAGGC
AA4031	GGATCCATGGATGCAGGGACT
AA4032	GGATCCAAGGCCAAGAAGGGAAA
AA4033	GGATCCAAGGGATTGCAAGTGGTCAG
AA1551	AGCTGTGCGTATCCAATAAT
AA3358	CTCCTCGCCCTTGCTCACCATCTTGGCCTTGCTGTTAAC
AA3357	GTTAACAGCAAGGCCAAGATGGTGAGCAAGGGCGAGGAG
AA1879	GCAAGAATTGGCTGCCCTCTAGGCATACCTGCCAAACCTAA
AA1880	TTAGGTTTGGCAGGTATGCCTAGAGGGCAGCCAATTCTTGC
AA1752	ACTGATAGATCTGAGGAAAGCGTCAAGGATATG
AA1463	AGTTCCGCATCAGTCTTGCT
AA1657	GAGGAAAGCGTCAAGGATATG
AA1489	CCTGAGGACGCTTGAAAGTC
AA3305	GGAGTTCGTGACCGCCGC
AA1753	AGTTAAGATCTATGGCTTACCCAATTCTCCC
AA3078	CAAGAATTGGCTGCCCTCTAAGTCGAGTTGGAAGAGAGTG
AA3079	AAGAGGGTCCACGCCTCCCTAGAGGGCAGCCAATTCTTGC
AA3491	CCTCGCCCTTGCTCACCATGAAGTAGTTGCCCTTGCCGGC
AA3492	GCCGGCAAGGGCAACTACTTCATGGTGAGCAAGGGCGAGG
AA3493	CAAAATAAAAAGAATGTATCAATCAGTACAGCTCGTCCATG
AA3494	CATGGACGAGCTGTACTGATTGATACATTCTTTTTATTTTG
AA4206	GCAGTACAGGTGGAGATTGC
AA4207	GTCATAGCTGTTTCCTGGTTGGCCTTGTTGCTTCA
AA4208	ACTGGCCGTCGTTTTACGGAGATAACCAACTCTTG
AA4209	CCCTCACTCTCAGATCGGTC
AA4210	GCCTCATCCAACGTCCTTTC
AA4211	GTCATAGCTGTTTCCTGGACTGTAATGGCCTTATG
AA4212	ACTGGCCGTCGTTTTACGGATCATGATGAAGGAGA
AA4213	TAAAGAGCTGGGTGTCTGGG
AA3934	TCGATGCGATGTTTCGCT
AA3935	CCTGAATGAACTGCAGGA
M13R	CAGGAAACAGCTATGAC
M13F -20	GTAAAACGACGGCCAGT

The *rim13Δ* (KS33), *rim23Δ* (KS94), *ali1Δ* (KS118), *ali2Δ* (KS94-2), *rim90* (KS63), *rim91* (KS57), *rim92Δ* (KS53) strains were created by replacing the entire open reading frame with the dominant selectable *NAT* or *NEO* resistance genes. Overlap PCR was used to create each KO cassette as previously described [61], which was introduced into the recipient strain by biolistic transformation [62]. The following primer combinations were used to create each KO construct: *RIM13* 5’ fragment: AA1796 and AA1792, *RIM13* 3’ fragment: AA1793 and AA1795, *NEO* resistance marker: AA1797 and AA1794. AA1796 and AA1795 were used to amplify the final deletion construct. *RIM23* 5’ fragment: AA3361 and AA3362, *RIM23* 3’ fragment: AA3365 and AA3366, *NEO* resistance marker: AA3363 and AA3364, AA3361 and AA3366 were used to amplify the final deletion construct. *ALI1* 5’ fragment: AA3254 and AA3255, *ALI1* 3’ fragment: AA3258 and AA3259, *NEO* resistance marker: AA3256 and AA3257. AA3254 and AA3259 were used to amplify the final deletion construct. *ALI2* 5’ fragment: AA3505 and AA3506 *ARR2* 3’ fragment: AA3509 and AA3510, *NEO* resistance marker: AA3507 and AA3508. AA3505 and AA3510 were used to amplify the final deletion construct. *RIM90* 5’ fragment: AA3267 and AA3268, *RIM90* 3’ fragment: AA3271 and AA3272, *NEO* resistance marker: AA3269 and AA3270. AA3267 and AA3272 were used to amplify the final deletion construct. *RIM91* 5’ fragment: AA3226 and AA3227, *RIM91* 3’ fragment: AA3230 and AA3231, *NEO* resistance marker: AA3228 and AA3229. AA3226 and AA3231 were used to amplify the final deletion construct. *RIM92* 5’ fragment: AA3260 and AA3261, *RIM92* 3’ fragment: AA3264 and AA3265, *NEO* resistance marker: AA3262 and AA3263. AA3260 and AA3265 were used to amplify the final deletion construct. The *ali1Δ ali2Δ* (KS97-2) strain was created by disrupting the *ALI2* gene in the KS118 strain. The *rra1Δ* (KS183) and *rra1Δ* + *eGFP-RIM101* (KS185) strain was created by using primers AA3970 and AA3971 to PCR amplify the *NEO* resistance marker from KS216 and introduce it into wild type H99. Each mutant strain was confirmed by Southern blot. The primers used to create each Southern probe were: *RIM13*: AA1812 and AA1813, *RIM23*: AA3401 and AA3402, *RIM90*: AA3292 and AA3267, *RIM91*: AA3241 and AA3242. *RIM92* AA3293 and AA3294, *ALI1*: AA3295 and AA3296. *ALI2*: AA3672 and AA3673, *RRA1*: AA4011 and AA4012.

The *C*. *gattii rim101Δ* and *rra1Δ* mutants were created in the R265 background using the split marker method previously described [63]. For the *Cgrim101Δ* mutant the following primers were used with R265 gDNA as the template. *CgRIM101* 5’ fragment: AA4206 and AA4207, *CgRIM101* 3’ fragment: AA4208 and AA4209. The 5’ region of the *NEO* cassette was amplified from pJAF using the M13R primer and AA3935 and the 3’ region with M13F -20 and AA3934. The *Cgrim101Δ* 5’ KO cassette was created by combining the 5’ *CgRIM101* and *NEO* PCR PCR products by overlap PCR using AA4206 and AA3935. The 3’ KO cassette was similarly made using AA3932 and AA4209. These overlap PCR products were combined and transformed into R265. The *Cgrra1Δ* mutant was created in the same way, using the following primer pairs. *CgRRA1* 5’ fragment: AA4210 and AA4211 and *CgRRA1* 3’ fragment: AA4212 and AA4213. Southern blot analysis was used to confirm a insertion at the correct locus.

The *GFP-RIM101T* strains were created by first creating TOC65 (*rim101Δ + GFP-RIM101T*). The following PCR primer combinations were used to amplify the *RIM101* promoter and terminator (from H99 gDNA) and a truncated *GFP-RIM101* fusion (from pTO2): *RIM101* promoter: AA1551 and AA3358, GFP-RIM101 truncation:AA3357 and AA1879, *RIM101* terminator: AA1880 and AA1752. Overlap PCR was then performed and was transformed, along with pJAF (*NEO*), into TOC2. Replacement of the *rim101Δ* NAT disruption construct was confirmed by PCR, and the expression and functionality of *GFP-RIM101T* was verified by reconstitution of the *rim101Δ* mutant phenotypes. Primers AA1463 and AA1657 were then used to amplify the promoter, ORF, and terminator of the GFP-70T from the TOC65 gDNA. The *GFP-RIM101T* was then co-transformed with either pJAF (*NEO*) or pCH233 (*NAT*) into the following mutant strains: *vps23Δ* (*vps23-9*), *rim13Δ* (KS33), *rim20Δ* (TOC17), *rim23Δ* (SK94). Transformants were screened using PCR (AA1489 and AA3305) and epifluorescence microscopy to detect the presence and expression of the *GFP-RIM101* fusion respectively.

KS289 (*rim23Δ*::*NEO* + *GFP-RIM23 + NAT MATα)* was created by creating a *RIM23-GFP* fusion using the following PCR reactions. AA3361 and AA3491 were used to amplify *RIM23* and its promoter from H99 gDNA. AA3492 and AA3493 were used to amplify GFP from a GFP-containing vector. AA3494 and AA3166 were used to amplify that *RIM23* terminator region. These PCR products were mixed and overlap PCR was performed using AA3361 and AA3366. The overlap piece was mixed with pCH233 (NAT) and transformed into KS94 (*rim23Δ)*. Transformants that had rescued growth on pH 8 all displayed GFP fluorescence. The isolate chosen for this study was rescued for 1.5 M NaCl growth and capsule formation. The other *RIM23-GFP* strains were created by crossing KS289 or KS290 (Mat**a**) to the each mutant strain. Progeny were selected based on pH 8 and 1.5M NaCl sensitivity and the genotypes were confirmed using PCR. *GFP-RIM23* was the only *RIM23* allele in each of these strains.

To reconstitute the *rim13Δ* mutant, primers AA1796 and AA1795 were used to amplify the *RIM13* promoter, gene, and terminator sequence and then co-transformed with pCH233 (*NAT*) into the KS33 strain. PCR and rescued phenotype were used to verify restored expression of *RIM13*. The *rim23Δ* mutant was similarly reconstituted using primers AA3361 and AA3366. The *rra1Δ* mutant was reconstituted by amplifying the *RRA1* promoter, ORF, and terminator using primers AA4033 and AA4032 and then TA cloning into pCR2.1. The insert was digested from the TA vector with BamHI cloned into the single BamHI site in pJAF to create pKS38. The KS183 strain (*rra1Δ*) was then transformed with pKS38 and transformants were screened for rescued mutant phenotypes.

The pKS34 vector was created by using AA4031 and AA4032 to amplify the *RRA1* gene from H99 genomic DNA and then TA cloned into pCR2.1. *RRA1* was then cloned into the single BamHI site in pCN19 [64].To create *rra1Δ* + *GFP-RRA1* (KS214), KS183 was transformed with pKS34.

The pTO22 construct was created by amplifying and truncating the *RIM101* gene using the following primer pairs: 5’ flank: AA1753 and AA1879, 3’ flank: AA1880 and AA1752. The entire piece was amplified using AA1753 and AA1752, TA cloned into pCR2.1, and then cloned into the single BamHI site in pCN70 (*pGAL7-GFP-NEO*).

### Insertional mutagenesis and assessment of mutants

*Agrobacterium tumefaciens-*mediated random mutagenesis was carried out as previously described [65]. Briefly, *A*. *tumefaciens* expressing the pPZP-NEOcc plasmid was incubated with shaking at room temperature for 48 hr in Luria-Bertani (LB) medium containing kanamycin. Cells were washed and resuspended to an OD_600_ of 0.15 in liquid induction media (IM) containing 200 μM acetosyringone (AS) and incubated for 6 hr An overnight YPD culture of KS161 was harvested, washed with IM and resuspended in IM at a concentration of 10^7^ cells/mL. 200 μL of the *A*. *tumefaciens* and KS161 suspensions were mixed and aliquoted on IM agar containing acetosyringone. Cells were incubated for 3 days and then scraped onto selection media (YPD + Neo + 100 μg/mL cefotaxime). Colonies passaged 1X on the selection plates and then inoculated into liquid selection media in 96-well plates. Duplicates of each isolate were inoculated. Mutants were then inoculated onto YP Dextrose or YP Galactose media containing 1.5M NaCl or 150mM HEPES adjusted to pH 8. A total of 3500 mutants were screened in duplicate. Mutants with growth defects on either condition were selected and rescreened. The genetic location of each insertion was identified by deep sequencing using MiSeq deep sequencing.

### Protein extraction, immunoprecipitation, and western blot

Protein extracts were prepared as previously described [66]. To initially assess GFP-Rim101 proteolytic processing at multiple pH’s, the *rim101Δ* + *GFP-RIM101* (TOC105) strain was grown overnight (~18 hr) in 50ml YPD, 30^°^C, 150 rpm shaking. Cells were pelleted and resuspended in SC McIlvaine’s buffer at pH 4, pH 6, or pH 8 to an OD_600nm_ of 1. Samples were further incubated at 30^°^C 150 rpm for 3 or 5 hr Cells were harvested, flash frozen on dry ice, and lysed in 0.4 mL lysis buffer containing 2x protease inhibitors (Complete, Mini, EDTA-free; Roche) and 1x phosphatase inhibitors (PhosStop; Roche) and 1 mM phenylmethanesulfonylfluoride (PMSF). Lysis was performed by bead beating (0.5 mL of 3 μM glass beads in a Mini-BeadBeater-16 (BioSpec), 10 cycles for 30 s each). Supernatants were transferred to new tubes and glass beads washed 2 times with 0.4 mL lysis buffer. Lysates were cleared by centrifugation at 15,000 rpm, 4^°^C, for 10min. 5 μLGFP-TRAP resin (Chemotek), which was equilibrated in lysis buffer, was added to the cleared lysate and inverted at 4^°^C for 4 hr GFP-TRAP resin was washed 3 times with 1 mL lysis buffer and protein was eluted in 30 μL NuPage sample buffer with 1X NuPage Reducing Agent by boiling for 5 min. Western blots were performed as described previously using 4–8% NuPage BisTris gels to separate the samples. To detect GFP-Rim101, blots were incubated in anti-GFP primary antibody (Roche) (using a 1/10,000 dilution) and then in secondary anti-mouse peroxidase-conjugated secondary antibody (using a 1/25,000 dilution, Jackson Labs).

Assessment of GFP-Rim101 proteolysis in the upstream Rim pathway mutants was performed as described above but with several alterations. First, YPD 150 mM HEPES adjusted to a pH 7.4 was used instead of SC McIlvaine’s buffer. Second, GFP-Rim101 was immunoprecipitated with anti-GFP antibody (Roche) for 1 hr and then incubated 2 hr with 80 μL Protein G Sepharose (Thermo Scientific). Finally, a 3–8% NuPage Tris-Acetate gel was used to separate samples.

### Microscopy

The high-resolution fluorescent images of Rim23-GFP were captured using a Delta Vision Elite deconvolution microscope equipped with a Coolsnap HQ2 high resolution charge-coupled-device (CCD) camera. All other differential interference (DIC) and fluorescent images were captured using a Zeiss Axio Imager A1 fluorescence microscope equipped with an AxioCam MRM digital camera.

### Virulence studies

Virulence studies were carried out according to [67]. 8–10 female A/Jcr mice were anesthetized with 100–140 μL of 12 mg/ml ketamine HCl and 1 mg/ml Xylazine in PBS. Each mouse was intranasally inoculated with 1X10^5^ cells in a volume of 25 μL. Mice were monitored and weighed daily and sacrificed based on predetermined symptoms that predict imminent death. Kaplan-Meyer curves were analyzed by log-rank test (JMP software; SAS Institute, Cary, NC). All studies were performed in compliance with Duke University institutional guidelines for animal experimentation.

### Ethics statement

All animal work was performed according to the Duke University Institutional guidelines for animal experimentation. All mice were anesthetized using an intraperitoneal injection of Ketamine/Xylazine mixture and were sacrificed by CO2. Approved secondary methods of ensuring animal death were also used. These techniques are consistent with IACUC-approved animal handling protocol- A217-11-08.

## Supporting Information

S1 Fig Rim101 proteolysis complex components. (A) Model of *C*. *neoformans* Rim13, Rim20, and Rim23 orthologs. Conserved protein domains were predicted using Pfam and Super Family databases. The E-values for each domain prediction: Rim23 PF03097: 5.50E-9; Rim20 PF03097: 1.90E-97, PF13949: 1.10E-71; Rim13 SSF54001: 1.16E-42, SSF49758 (2 domains): 1.57E-19 and 2.09E-14. (B) Expression of *RIM13* and *RIM23* wild type alleles rescues *rim13Δ* and *rim23Δ* pH 8s and 1.5 M NaCl growth defects. (C) Expression of the wild type alleles also rescues the *rim13Δ* and *rim23Δ* mutant capsule defects. Cells were cultured for 48 hr in CO_2_-independent media 37^°^C to induce capsule formation. Capsule was visualized by India ink stain.
(TIF)

S2 Fig Predicted arrestins and Rim9 orthologs are not required for Rim101-dependent phenotypes. (A) The *ali1Δ*, *ali2Δ*, *ali1Δ ali2Δ*, and *rim90Δ rim91Δ rim92Δ* mutants grow like WT on YPD with 150 mM HEPES at pH 8 and YPD + 1.5 M NaCl. 10-fold serial dilutions of each sample were spotted onto the indicated plates. (B) The *ali1Δ*, *ali2Δ*, *ali1Δ ali2Δ*, and *rim90Δ rim91Δ rim92Δ* mutants do not have a capsule formation defect. Cells were cultured for 48 hr in CO_2_-independent media 37^°^C to induce capsule formation. Capsule was visualized by India ink stain.
(TIF)

S3 Fig Alignment of the conserved regions of the basidiomycete Rra1 orthologs. The following orthologs are represented: *C*. *neoformans* CNAG_03488 (Rra1), *C*. *gattii* CGB_G5320C, *Tremella mesenterica* TREME_69388, *Puccinia graminis* PGTG_03106, *and Ustilago maydis* um00299. The gray shaded regions mark predicted transmembrane helices. The similarity between sequences is represented by color, with red representing the most similar and blue representing the least similar. Alignment was created using the T-Coffee multiple sequence alignment server [68]. The numbers indicate amino acid position.
(TIF)

S4 Fig Role of Gpa proteins in pH 8 and 1.5 M NaCl tolerance. The indicated strains were spotted onto YPD, YPD 150mM HEPES pH 8, and YPD 1.5M NaCl and incubated for 2–4 days at 30^°^C.
(TIF)
