# Supplementary figures and images for: The Cryptococcus neoformans Alkaline Response Pathway: Identification of a Novel Rim Pathway Activator
Source: PLoS Genet. 2015 Apr 10;11(4):e1005159. doi: 10.1371/journal.pgen.1005159 (PMC4393102; doi:10.1371/journal.pgen.1005159)

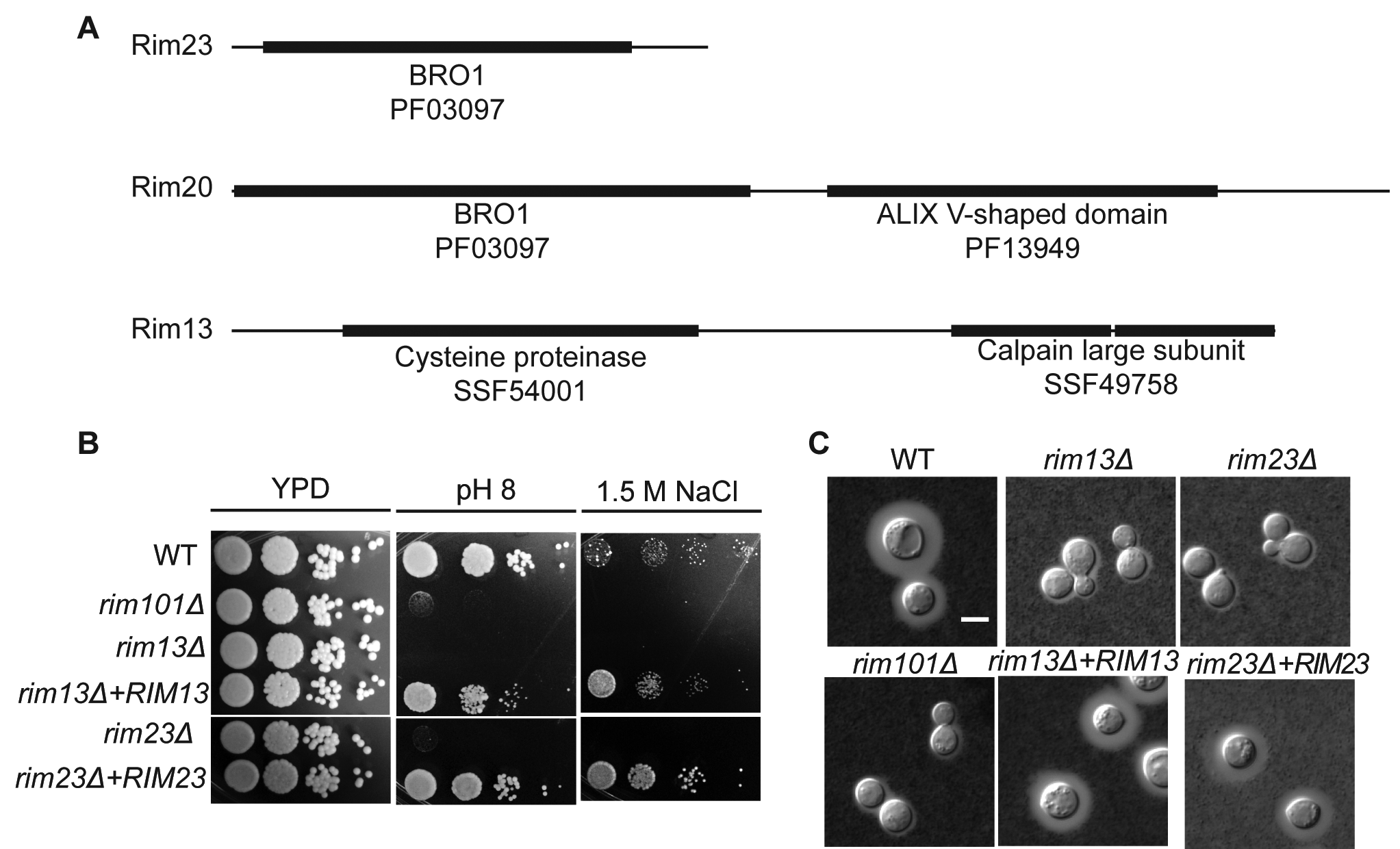

Supplement: S1 Fig — (A) Model of C. neoformans Rim13, Rim20, and Rim23 orthologs. Conserved protein domains were predicted using Pfam and Super Family databases. The E-values for each domain prediction: Rim23 PF03097: 5.50E-9; Rim20 PF03097: 1.90E-97, PF13949: 1.10E-71; Rim13 SSF54001: 1.16E-42, SSF49758 (2 domains): 1.57E-19 and 2.09E-14. (B) Expression of RIM13 and RIM23 wild type alleles rescues rim13Δ and rim23Δ pH 8s and 1.5 M NaCl growth defects. (C) Expression of the wild type alleles also rescues the rim13Δ and rim23Δ mutant capsule defects. Cells were cultured for 48 hr in CO2-independent media 37°C to induce capsule formation. Capsule was visualized by India ink stain. (TIF) [file pgen.1005159.s001.tif]

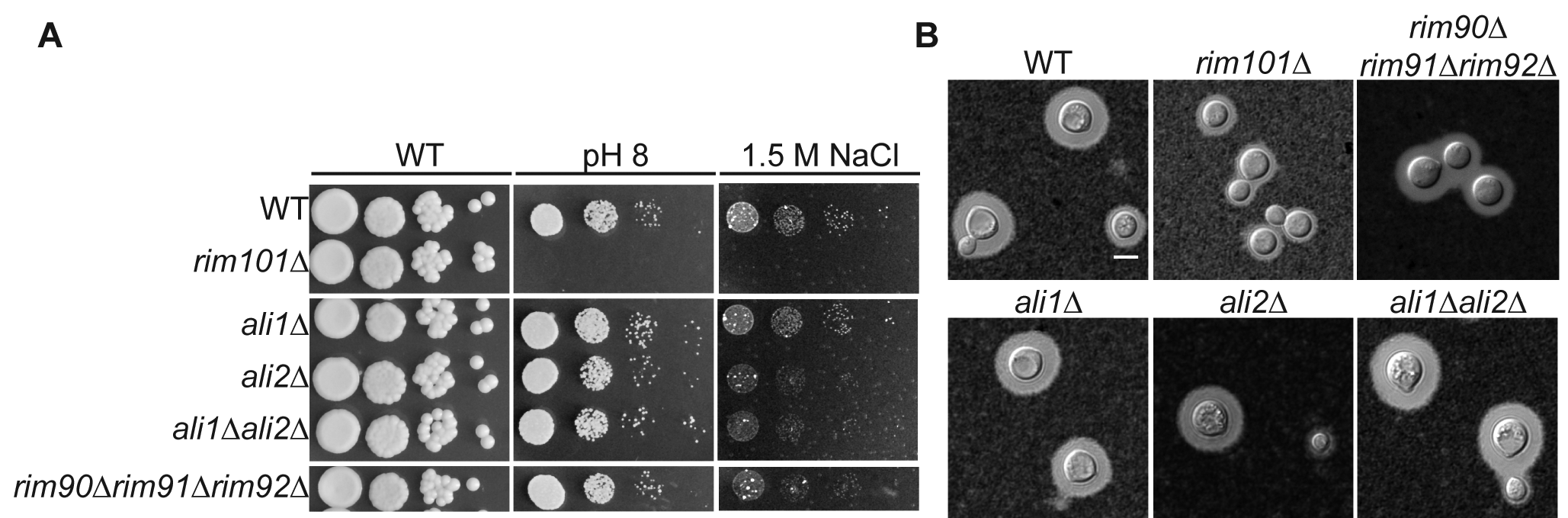

Supplement: S2 Fig — (A) The ali1Δ, ali2Δ, ali1Δ ali2Δ, and rim90Δ rim91Δ rim92Δ mutants grow like WT on YPD with 150 mM HEPES at pH 8 and YPD + 1.5 M NaCl. 10-fold serial dilutions of each sample were spotted onto the indicated plates. (B) The ali1Δ, ali2Δ, ali1Δ ali2Δ, and rim90Δ rim91Δ rim92Δ mutants do not have a capsule formation defect. Cells were cultured for 48 hr in CO2-independent media 37°C to induce capsule formation. Capsule was visualized by India ink stain. (TIF) [file pgen.1005159.s002.tif]

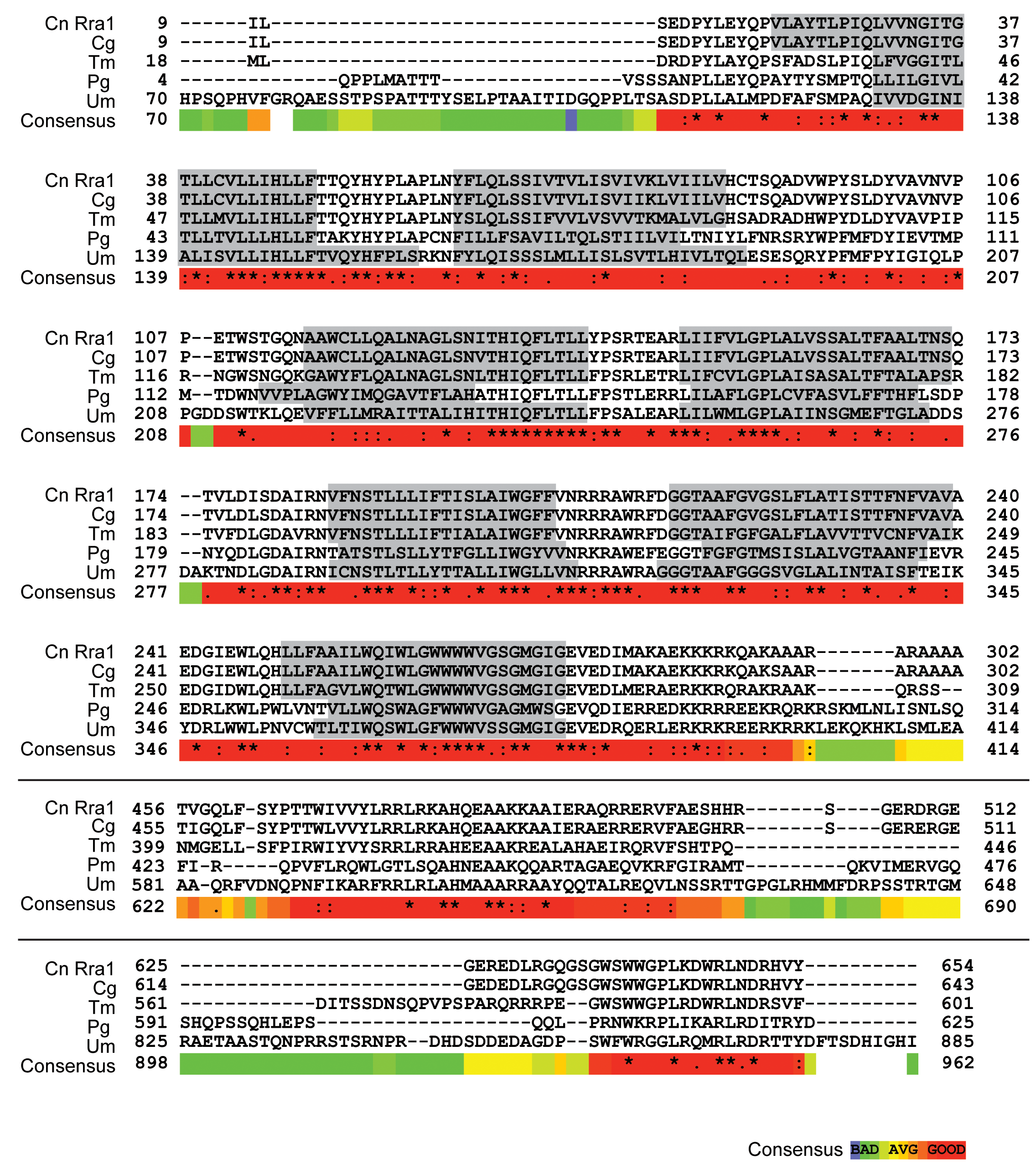

Supplement: S3 Fig — The following orthologs are represented: C. neoformans CNAG_03488 (Rra1), C. gattii CGB_G5320C, Tremella mesenterica TREME_69388, Puccinia graminis PGTG_03106, and Ustilago maydis um00299. The gray shaded regions mark predicted transmembrane helices. The similarity between sequences is represented by color, with red representing the most similar and blue representing the least similar. Alignment was created using the T-Coffee multiple sequence alignment server [68]. The numbers indicate amino acid position. (TIF) [file pgen.1005159.s003.tif]

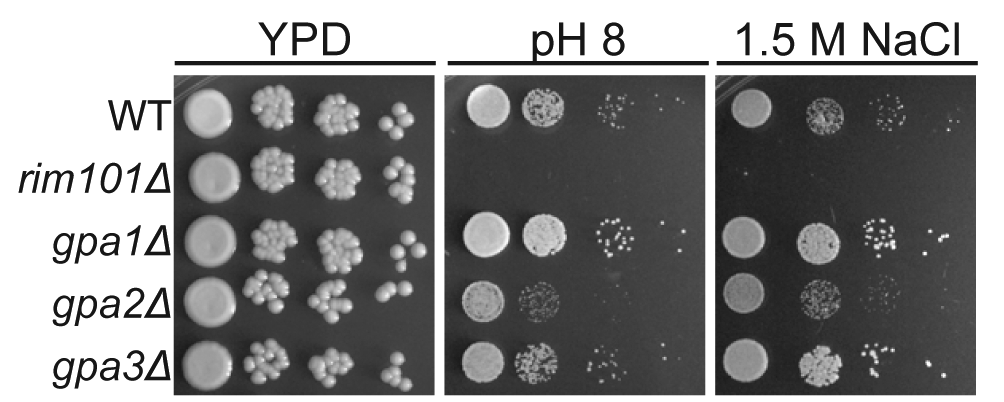

Supplement: S4 Fig — The indicated strains were spotted onto YPD, YPD 150mM HEPES pH 8, and YPD 1.5M NaCl and incubated for 2–4 days at 30°C. (TIF) [file pgen.1005159.s004.tif]
